# Study on the earth pressure of a foundation pit adjacent to a composite foundation with rigid-flexible and long-short piles

**DOI:** 10.1371/journal.pone.0251985

**Published:** 2021-05-20

**Authors:** Yuancheng Guo, Shaochuang Gu, Junwei Jin, Mingyu Li

**Affiliations:** School of Civil Engineering, Zhengzhou University, Zhengzhou, Henan, China; Mirpur University of Science and Technology, PAKISTAN

## Abstract

Model tests were performed to investigate the lateral earth pressure acting on the retaining structure adjacent to both natural ground (NG) and composite foundation (CFRLP), which were supported with rigid-flexible and long-short piles. Two testing procedures, namely, applying a load to the foundation and rotating the retaining structure along its toe, were considered. The results indicate that the additional lateral earth pressure acting on the retaining structure adjacent to the CFRLP is less than that of the NG in the depth of the reinforcement area strengthened by flexible piles. Compared with NG, the CFRLP yielded a smaller normalized height of application of the lateral earth pressure, suggesting that the CFRLP blocked the horizontal diffusion of the load and had a strong ability to transfer the surcharge load to the deep soil. When rotating the retaining structure, the lateral earth pressure acting on the upper part of the retaining structure experienced limited reduction once the displacement at the top of the retaining structure was greater than 8 mm, whereas the pressure acting on the lower part of the retaining structure continued to decrease with increasing displacement. In addition, a three-dimensional finite element model (FEM) was used to investigate the influence of the pile parameter and the wall-soil friction angle on the additional lateral earth pressure.

## 1. Introduction

Due to urbanization and space limitations, new pits will inevitably be excavated near existing high-rise buildings that were built on composite foundation with rigid-flexible and long-short piles (CFRLP). The existence of rigid-flexible and long-short piles will affect the lateral earth pressure acting on the retaining structure of the pits. It is critical to understand the lateral earth pressure due to such excavation to assess the serviceability and failure risk of the retaining structure.

Typical composite foundations include cushions and long-short piles. Short piles are applied to improve the bearing capacity of shallow soil, and long piles are placed into deep soil to control settlement [1–7]. The cushion between the raft and piles adjusts the load-sharing ratio among piles and mobilizes the bearing capacity of the soil. Some research has been performed to investigate the performance of composite foundations. Chen et al. [8] monitored the settlement of a 14-story building and obtained the pile-soil interaction mechanism in a composite foundation. The authors believed that flexible piles could accelerate the consolidation of soft soil, and due to the existence of the cushion, the bearing capacity of the subsoil would be fully mobilized. Zheng et al. [9] numerically investigated the response of composite foundations to various surcharge loads. The authors claimed that the length of the pile in the composite foundation has significant effects on the settlement of the foundation and that the greater the length of the pile, the less the settlement is. Sharma et al. [10] numerically investigated the main factors influencing the settlement of composite foundations. The authors found that the settlement of the raft decreases with an increase in the cushion thickness. Currently, composite foundations have been widely used in China.

Knowledge of the magnitude and distribution of lateral earth pressures acting on retaining structures is critical to the design of foundation pits. Many scholars have investigated the lateral earth pressure when the retaining structure is displaced. Bang [11] described a simple analytical method to predict the active lateral earth pressure under various amounts of wall displacement. Fang et al. [12] experimentally studied the active earth pressure when rotating a wall around the top of the retaining structure. The results showed that the stress distribution is nonlinear along the wall, and due to the arching effect, the stress near the top of the wall would increase beyond the level of the at-rest stress. Paik et al. [13] proposed a method to calculate the active earth pressure when the retaining wall was displaced. This method considered the principal stress deflection caused by the arching effect. Based on Paik’s theory, Goel et al. [14] investigated the distribution of active earth pressure and the shape of the critical failure surface. The authors found that a planar failure surface with a parabolic arch shape effectively predicts the experimental results. Chang [15] presented a simple calculation method that considered the deformation pattern and the associated mobilization of the shear resistance in soil. This method was successfully used to predict the lateral pressures for walls rotating about their base. Rao [16] proposed a new simplified method to calculate the active earth pressure acting on the retaining wall under translation mode. This method considered the effect of soil arching and friction mobilized along the wall–soil interface. Khosravi et al. [17] conducted a series of physical model tests to investigate the active earth pressure of a rigid retaining wall subjected to horizontal translation. The authors found that once the wall movement reached the so-called active wall movement, the earth pressure would reach a nearly constant value and would not change with further wall movement. Li et al. [18] proposed a new method to calculate the active earth pressure acting on a rigid retaining wall that moved horizontally away from the soil mass. This method considered that the soil arching effect induced the trajectory of the minor principal stress. Among those studies [11–18], both analytical methods and model tests have been conducted to investigate the active earth pressure acting on the retaining wall with displacement. However, the ground is normally considered to be natural ground (NG) without any reinforcement. There are very limited studies that consider the earth pressure on retaining walls adjacent to composite foundations.

The interaction between existing buildings and foundation pits has also been investigated in many studies. Finno et al. [19] described the performance of piles adjacent to excavation. The authors claimed that the observed movements were correlated with the excavation process. Liang et al. [20] developed a method based on Mindlin’s solution to analyze the shielding effect between piles. The parametric study showed that the shielding effect was governed by the axial load applied on piles. Poulos et al. [21] studied pile response due to excavation-induced lateral soil movements using the finite element method and the boundary element method. The authors found that the pile head condition had a major effect on the pile bending movement. Tong et al. [22] conducted a series model test to study the lateral earth pressures of NG and composite foundations. The author found that the lateral earth pressure of composite foundations is smaller than that of NG, and the distribution range of the lateral earth pressure of composite foundations is larger than that of NG. Korff et al. [23] proposed an analytical model to describe the pile deformation induced by deep excavation. The author found that the lateral pile response was mainly affected by the relative stiffness of the pile on the soil. Ong [24, 25] conducted a series of centrifuge model tests to investigate the behavior of a single pile subjected to excavation-induced soil movements. The authors found that after the completion of soil excavation, the wall and the soil continue to move, and such movement induces a further bending moment and deflection on an adjacent pile. Nishanthan et al. [26] investigated the shielding effect within piles adjacent to deep excavations. The authors found that the presence of front piles tends to significantly reduce excavation-induced movements. Shakeel et al. [27] conducted a three-dimensional analysis to gain insight into the response of pile groups adjacent to deep excavation. The authors found that the mobilization of shaft resistance along the pile depth highly depends on the relative pile toe position with respect to the excavation level. The above-mentioned studies provide additional insight into the interaction between the excavation and adjacent buildings [19–27]. However, the earth pressure acting on the retaining structure adjacent to the CFRLP was not considered in the authors’ work.

**Fig 1:**
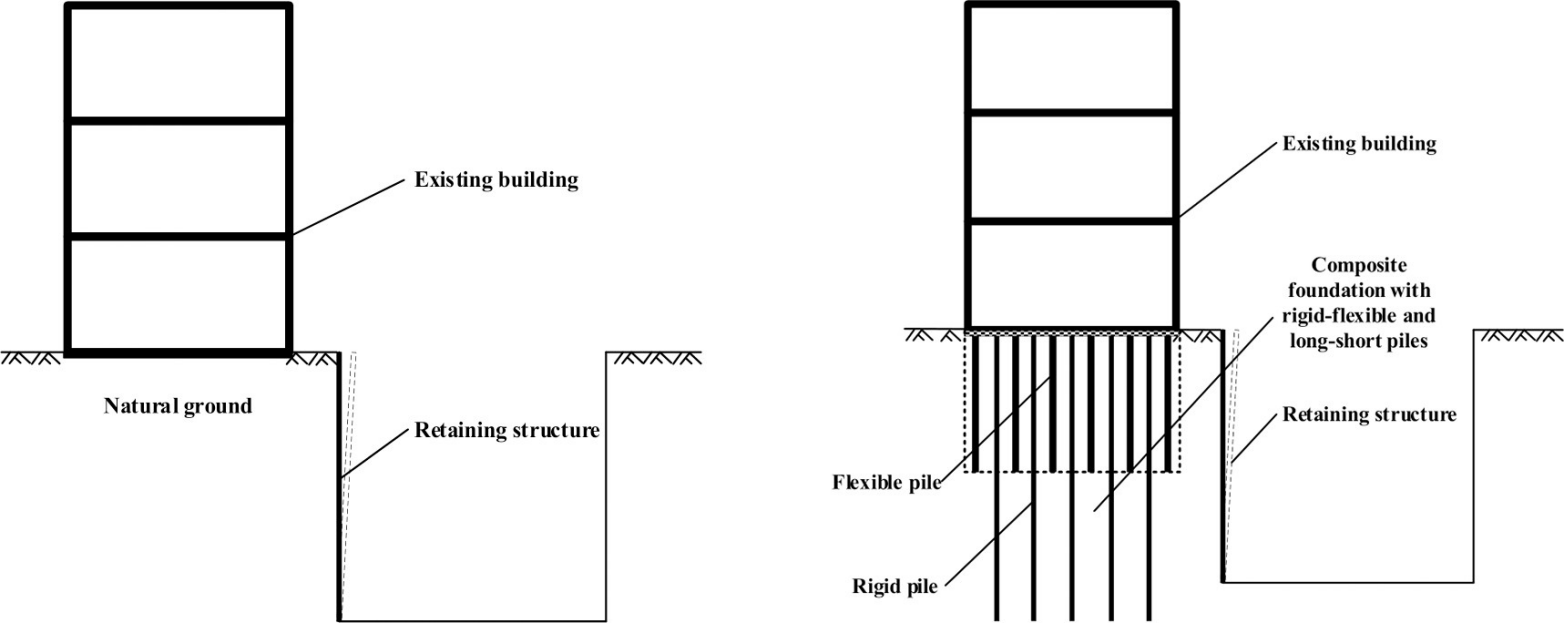
Relationship between the existing building and foundation pit. (a) Foundation pit adjacent to NG. (b) Foundation pit adjacent to the CFRLP.

In this study, two sets of model tests were performed to investigate the distribution and evolution of the earth pressure acting on the retaining structure adjacent to both NG and the CFRLP (see Fig 1). Each set of tests was conducted under two working conditions: applying surcharge loads to the foundation and rotating the rigid retaining structure. The evolution of the earth pressure acting on the retaining structure adjacent to the CFRLP was monitored and discussed. In addition, a three-dimensional finite element model (FEM) was used to analyze the lateral earth pressure, and the effect of the parameters of the flexible pile were investigated.

## 2. Experimental scheme

### 2.1 Test apparatus

**Fig 2:**
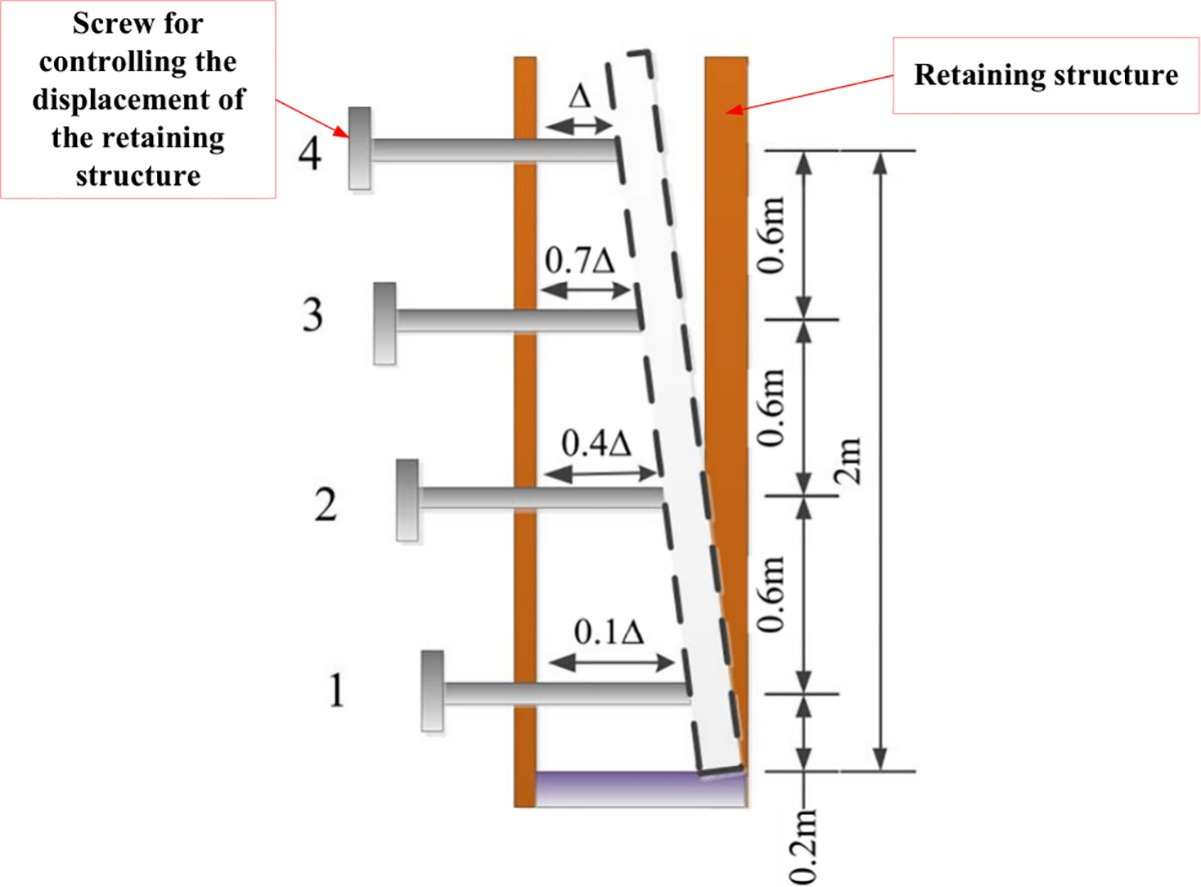
Schematic diagram of the rotation of the retaining structure.

**Fig 3:**
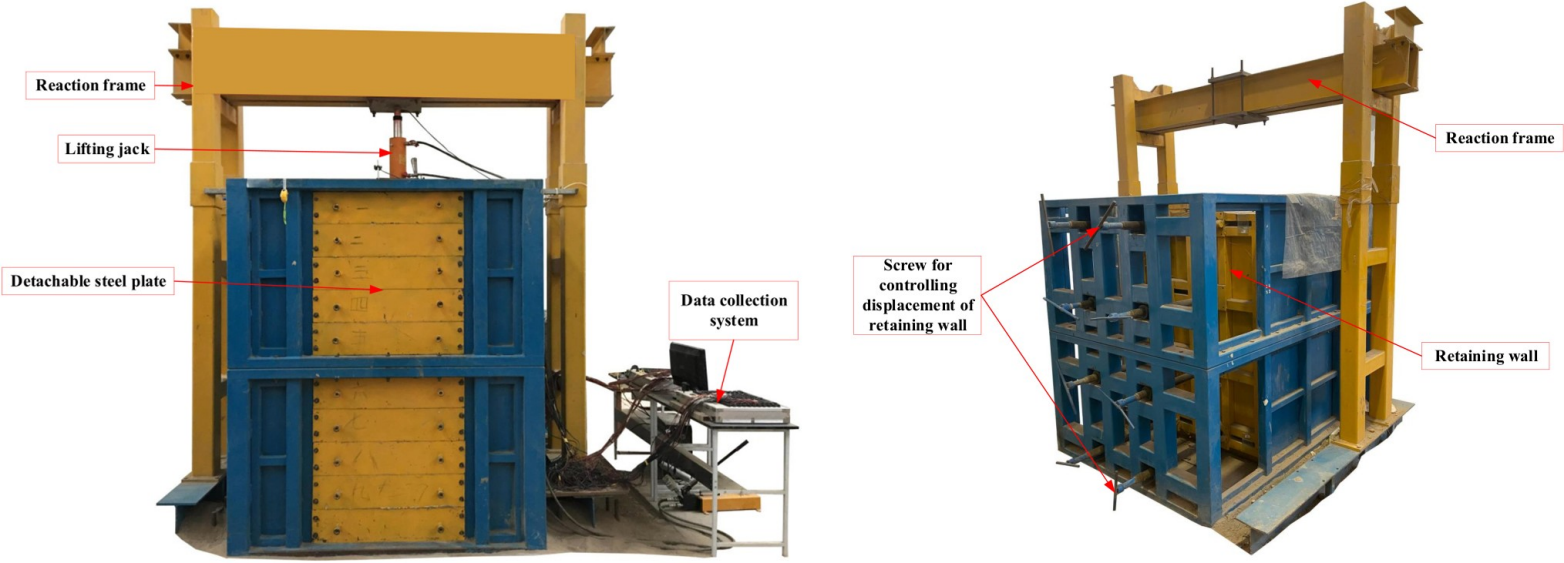
Annotated photos of the model box. (a) Front of the model box. (b) Back of the model box.

A model box with an internal compartment of 1.6 m (length) × 1.6 m (width) × 2.7 m (height) was used in the test. The box had 10 detachable steel plates on the front to facilitate the addition and removal of soil. The backside of the box consisted of a movable retaining structure with a height of 2 m. The outside of the retaining structure was a steel frame with screws. The ends of the screws were connected to the movable retaining structure. The displacements of the retaining structure at different heights were produced by rotating the screws on the steel frame. A schematic diagram of the retaining structure and its displacement control is shown in Fig 2. The surcharge load was provided by a combination of a jack and the reaction frame, as illustrated in Fig 3.

### 2.2 Soil properties

**Fig 4:**
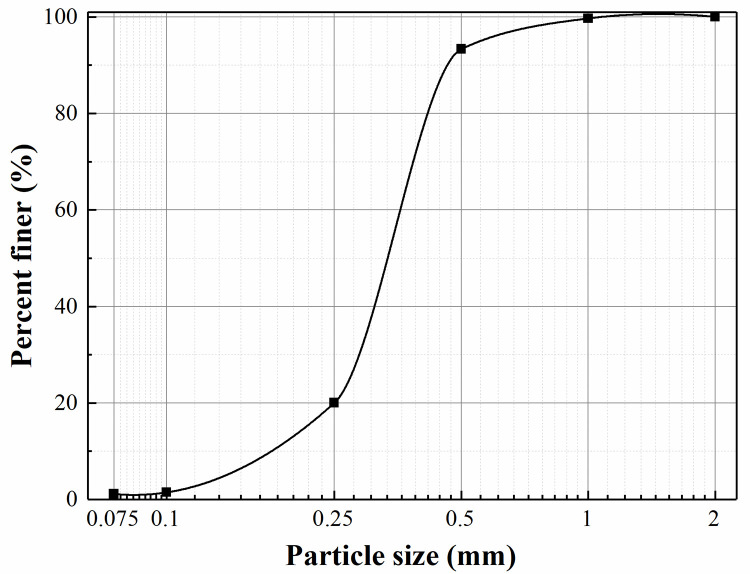
Particle-size distribution curve of the backfill.

**Fig 5:**
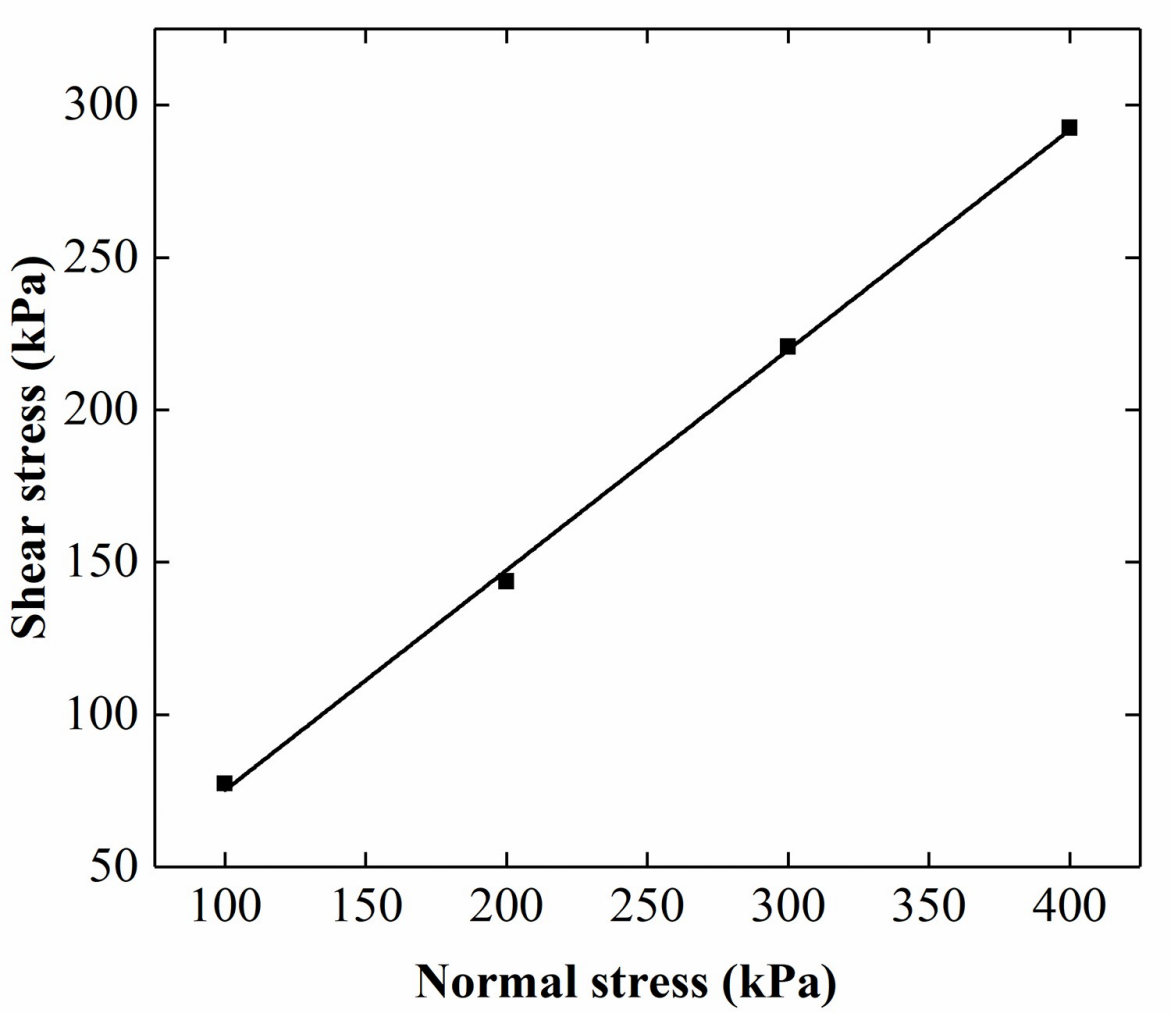
Direct shear test results of the sand.

**Fig 6:**
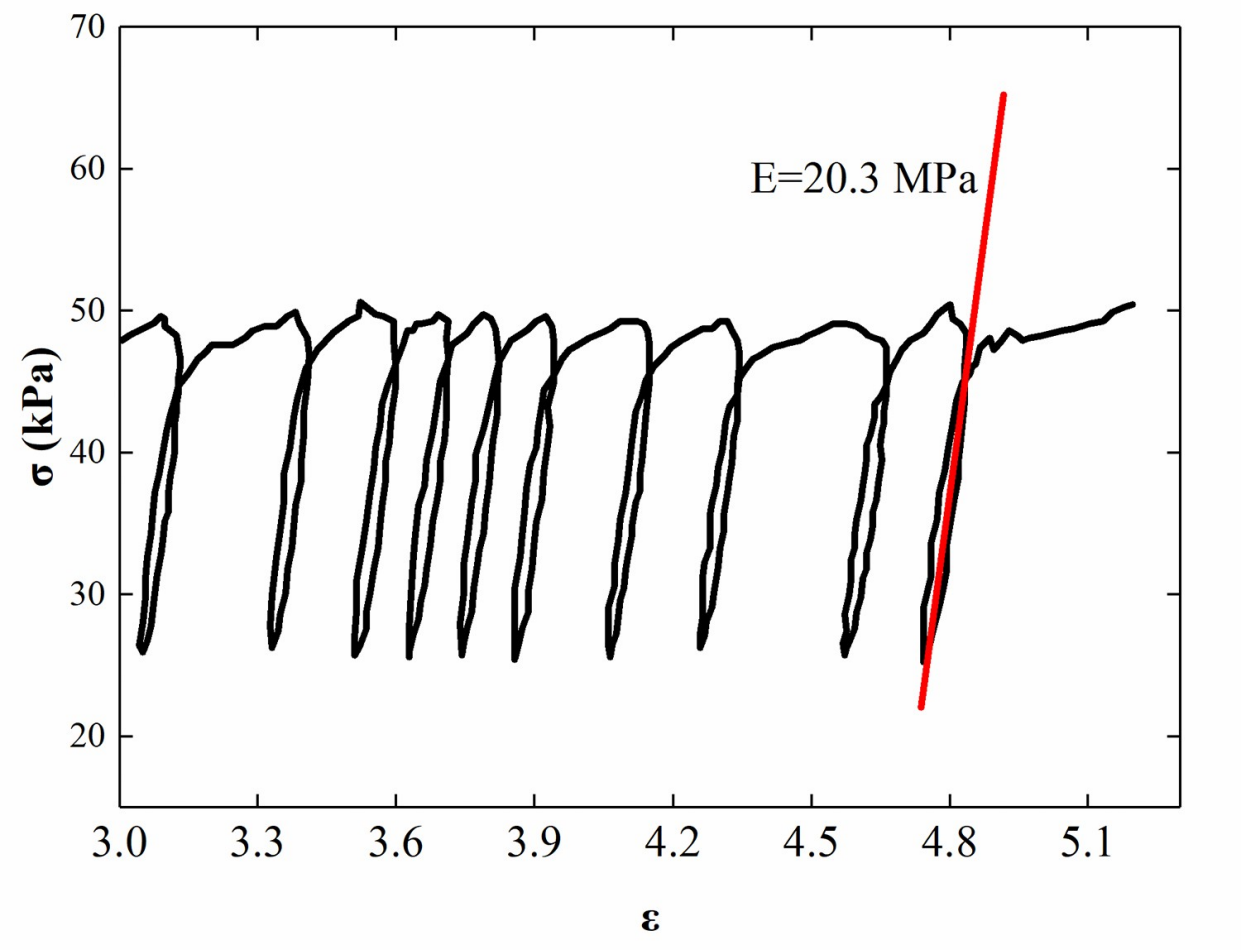
Triaxial test results of the sand.

The backfill used in this test was air-dried sand, and the properties of this sand are listed in Table 1. The particle-size distribution curve of the backfill is shown in Fig 4. The internal friction angle of soil is obtained by the direct shear test (see Fig 5). In reference to Zhou’s research [28], the elastic modulus of sand was determined to be 20.3 MPa by triaxial test (see Fig 6).

**Table 1:** Basic properties of the backfill.

Property	Density *ρ*: kg/m^3^	Moisture content *ω*: %	Internal friction angle *φ*: degree	Elastic modulus E: MPa
Value	1161	0.21	36.2	20.3

### 2.3 Material of the model pile

**Fig 7:**
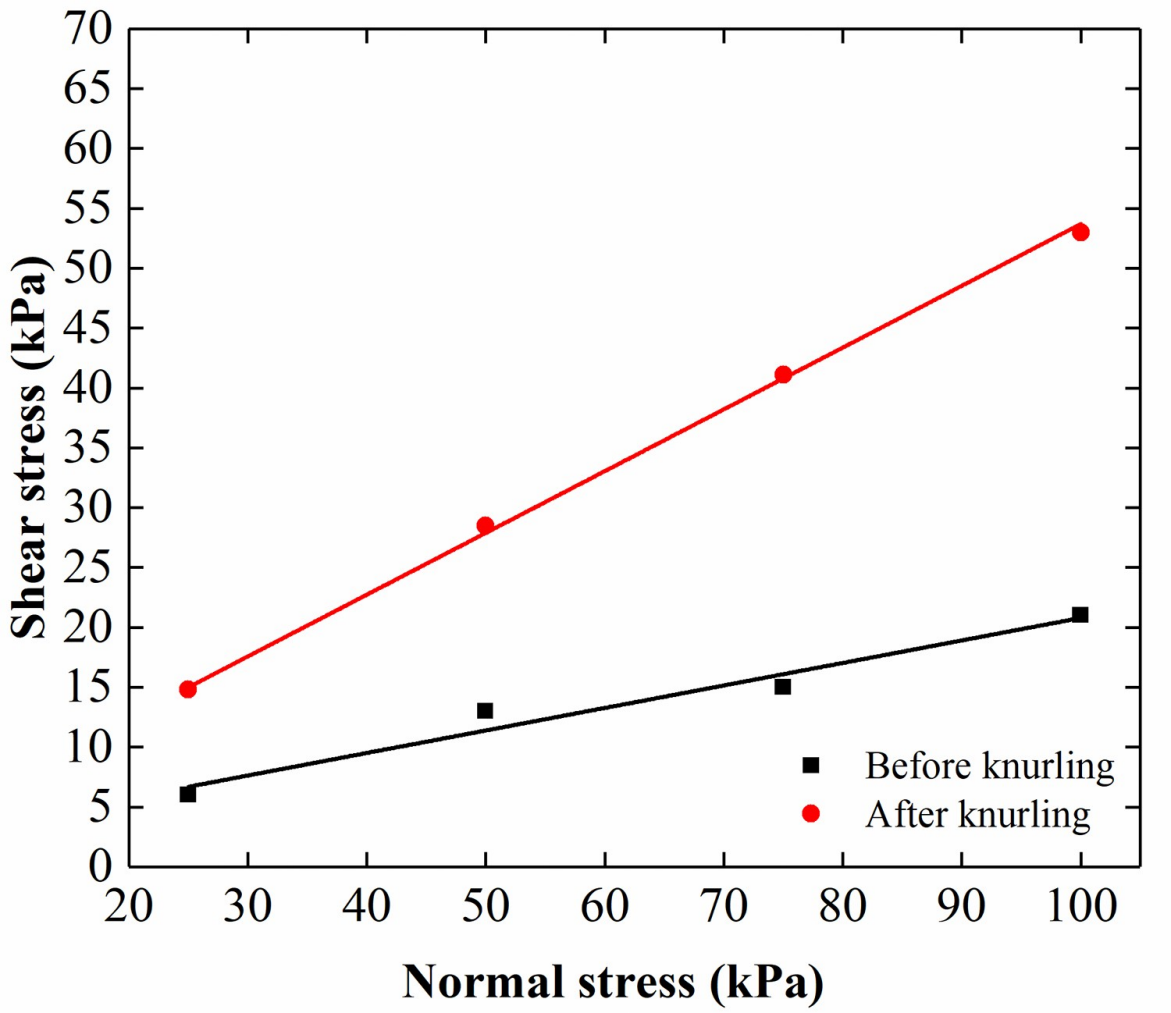
Direct shear test results from the interface of the rigid pile-soil.

**Fig 8:**
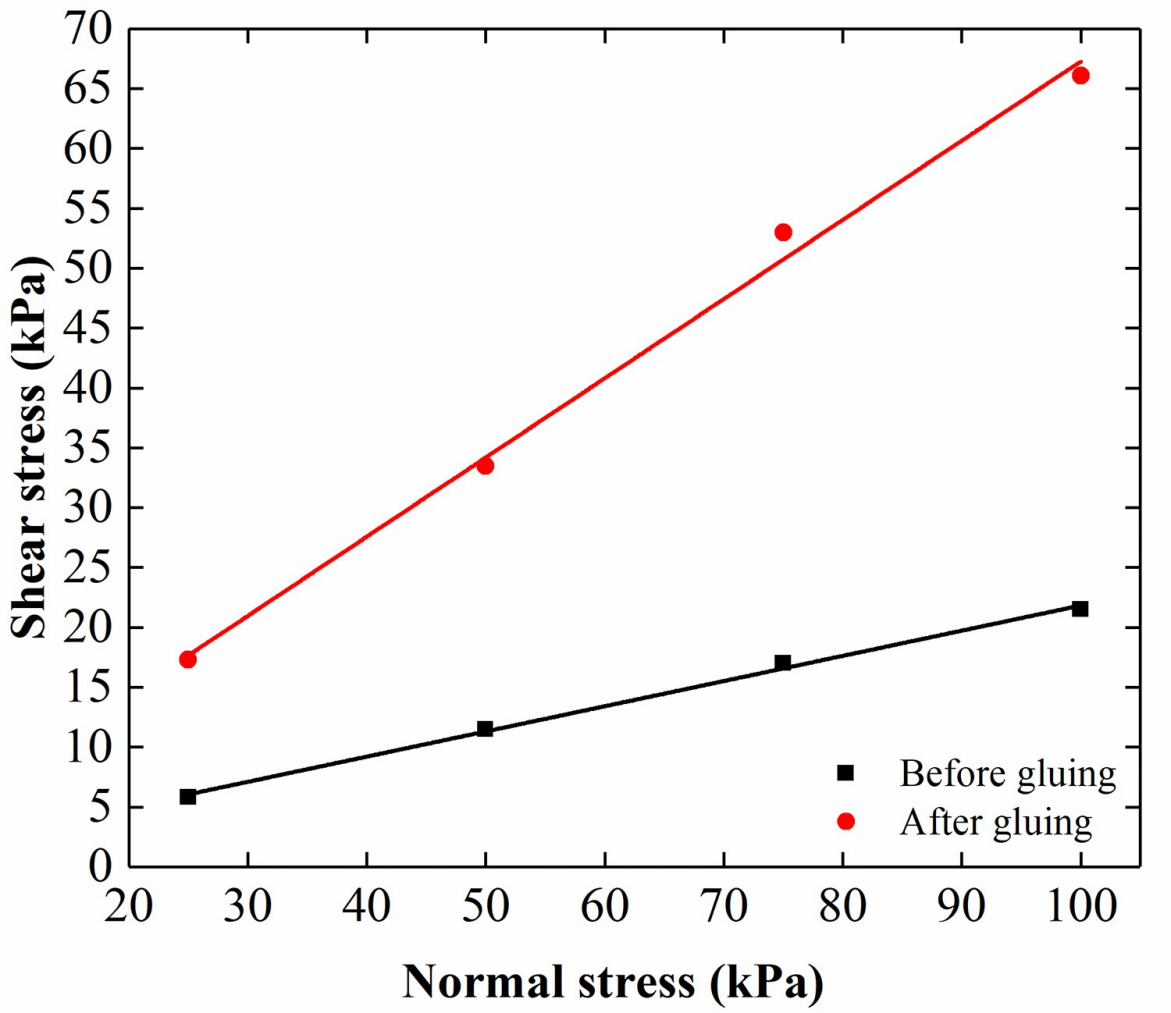
Direct shear test results from the interface of the flexible pile-soil.

**Fig 9:**
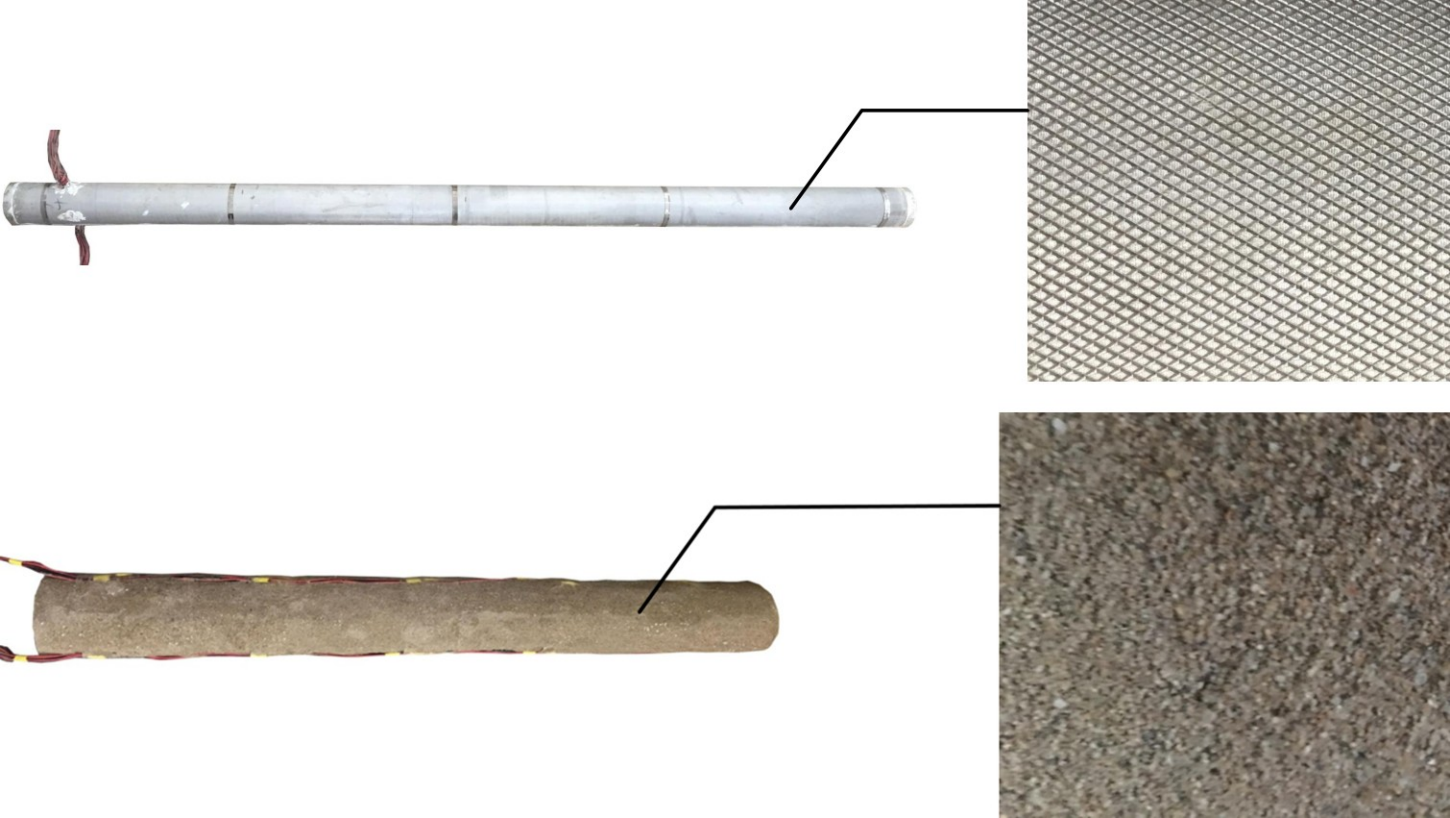
Photographs of the model pile. (a) Rigid pile. (b) Flexible pile.

The rigid pile was simulated using aluminum tubes with a diameter of 100 mm and a thickness of 2 mm. The length and elastic modulus of the rigid pile were 2.1 m and 13.68 GPa, respectively. The outside of the aluminum tube was knurled to increase the surface roughness and the friction between the model pile and the sand. The direct shear test (see Fig 7) shows that the pile-soil friction angle of long pile changed from 10.6° to 27.3° after knurling. A polyurethane rubber with a diameter of 120 mm and a length of 1 m was used to simulate the flexible pile. The elastic modulus of the pile was 60.35 MPa. Sand was glued to the surface of the pile to increase the friction between the pile and the sand. The direct shear test (see Fig 8) shows that the pile-soil friction angle changed from 11.9° to 33.5° after the sand was glued to the pile. Photographs of the rigid and flexible piles are shown in Fig 9.

According to previously reported research [29, 30], the pile with a pile-soil relative stiffness ratio of less than 1 is a flexible pile and the pile with a pile-soil relative stiffness ratio of more than 1 is a rigid pile. The pile-soil relative stiffness ratio is calculated as follows:

$$K=\sqrt{\frac{2E(1+\upsilon_{s})}{E_{s}}}\frac{D}{L}$$

(*K* is the pile-soil relative stiffness ratio; *D* is the diameter of the pile; *L* is the length of the pile; *E* is the elastic modulus of the pile; *E_s_* is the elastic modulus of soil; and *υ_s_* is Poisson’s ratio).

After the calculation, the pile-soil relative stiffness ratios of the short pile and long pile in this model test are 0.33 and 4.18, respectively. Therefore, the short and long piles used in this model test are flexible and rigid piles, respectively.

### 2.4 Layout of the sensors and piles

**Fig 10:**
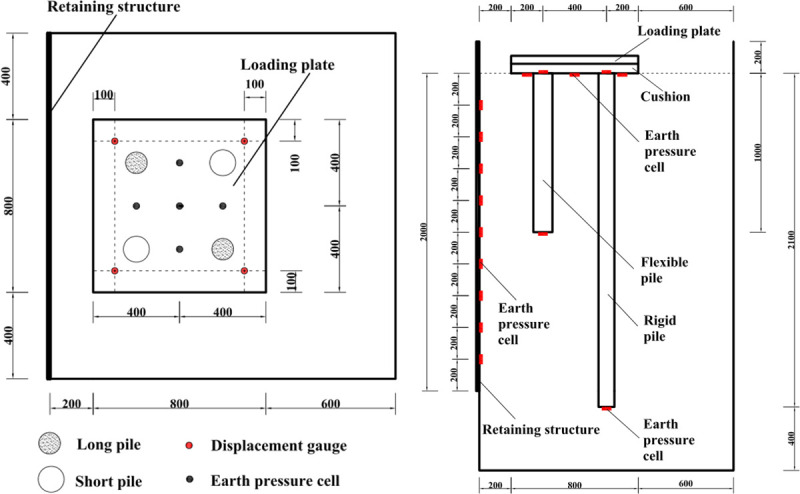
Diagram of the CFRLP and sensors (unit: mm). (a) Planform. (b) Side view.

In the model test, the plane size of the loading plate was 0.8 m (length) × 0.8 m (width). The distance between the edge of the loading plate and the movable retaining structure was 0.2 m, and the size of the cushion was 0.8 m (length) × 0.8 m (width) × 0.06 m (thick). Two rigid piles and two flexible piles were located under the cushion. The diameters of the earth pressure cells were 350 mm and 1000 mm, respectively. Each pile was equipped with two earth pressure cells (with a diameter of 1000 mm) to monitor the earth pressure at both ends of the pile. Five earth pressure cells (with a diameter of 350 mm) were placed in the backfill to monitor the vertical pressure between the piles. Nine earth pressure cells (with a diameter of 350 mm) were embedded in the midline of the movable retaining structure to monitor the lateral earth pressure. The distance between the earth pressure cells on the retaining structure was 200 mm. The details of the layout of the piles and monitoring instruments are shown in Fig 10.

**Fig 11:**
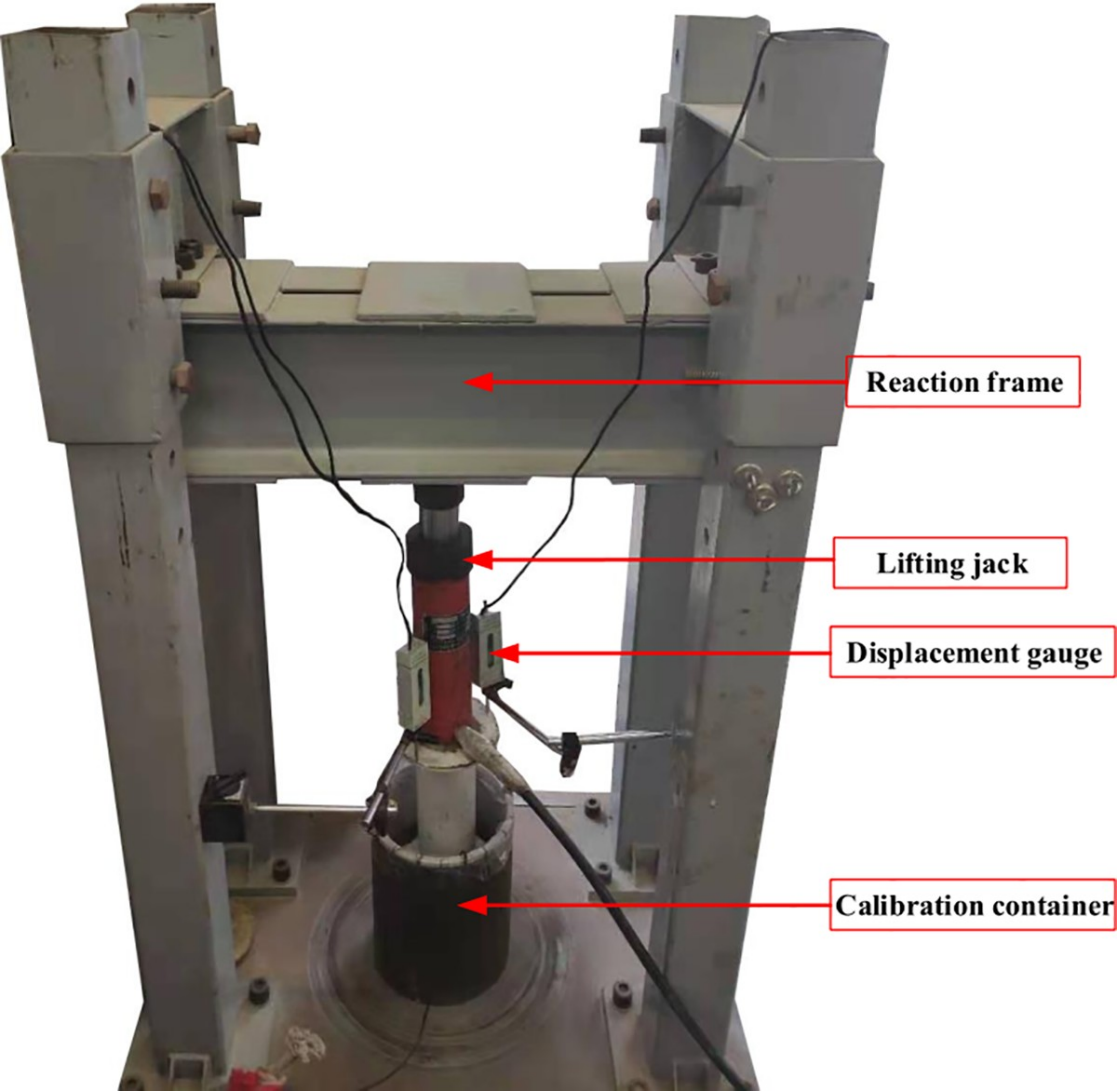
The photograph of the calibration devices.

To reduce the measurement error caused by the earth pressure cells, it is necessary to calibrate the cells to make them work properly in different testing environments. Compared with calibration with a liquid, calibration of the earth pressure cells with sand can reduce the measurement error caused by the soil arching effect and nonuniform contact stress. In this paper, sand was used to calibrate the earth pressure cells. An earth pressure cell can be divided into two basic categories [31] based on application: embedment and contact. Embedment cells are installed within the soil to measure the earth pressure of the soil between piles. Contact cells are used for measuring the lateral earth pressures acting on the retaining structure. The two types of earth pressure cells are calibrated separately in the calibration test of this study. The photograph of the calibration devices is shown in Fig 11.

### 2.5 Test procedure

**Fig 12:**
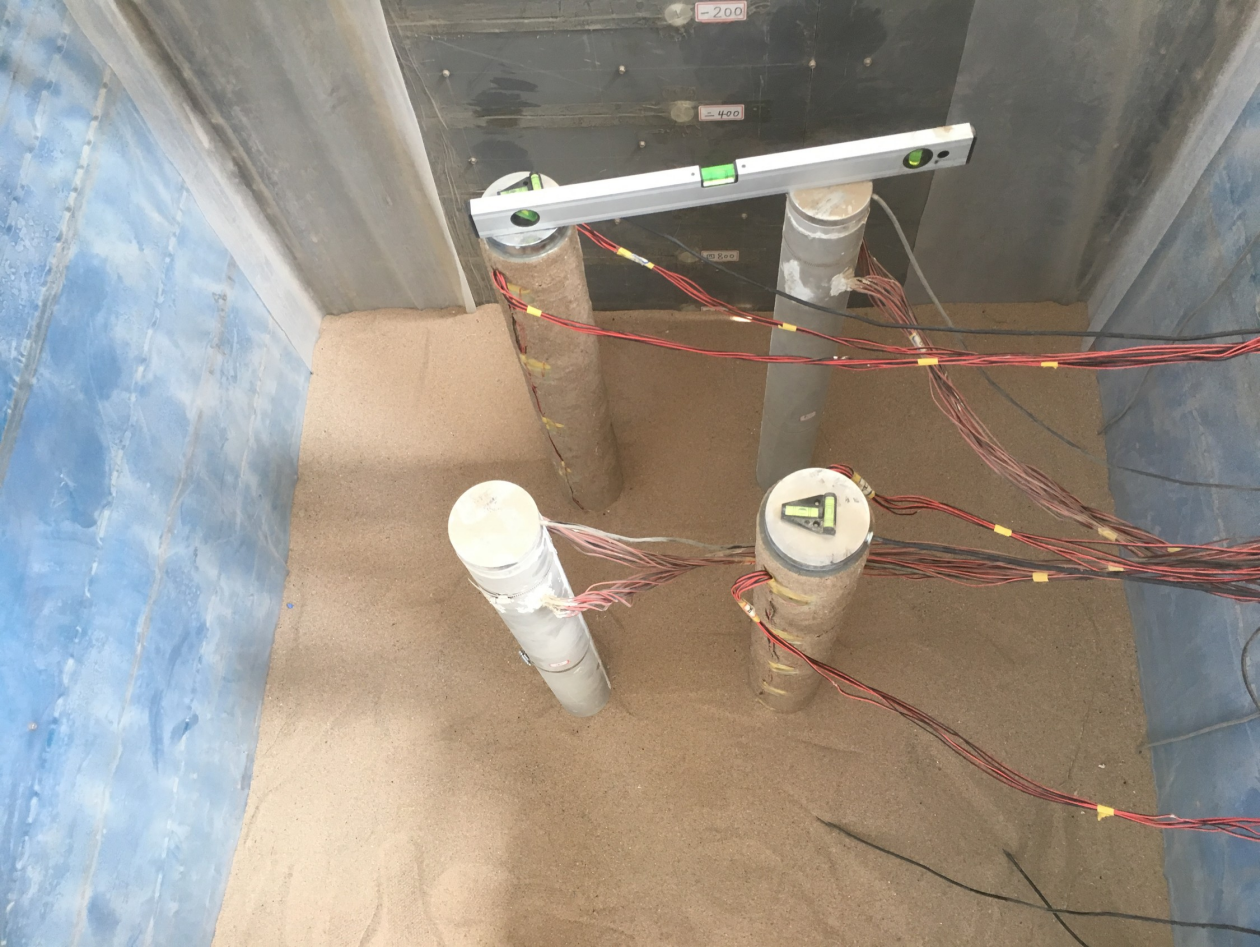
Photograph of installing the piles.

**Fig 13:**
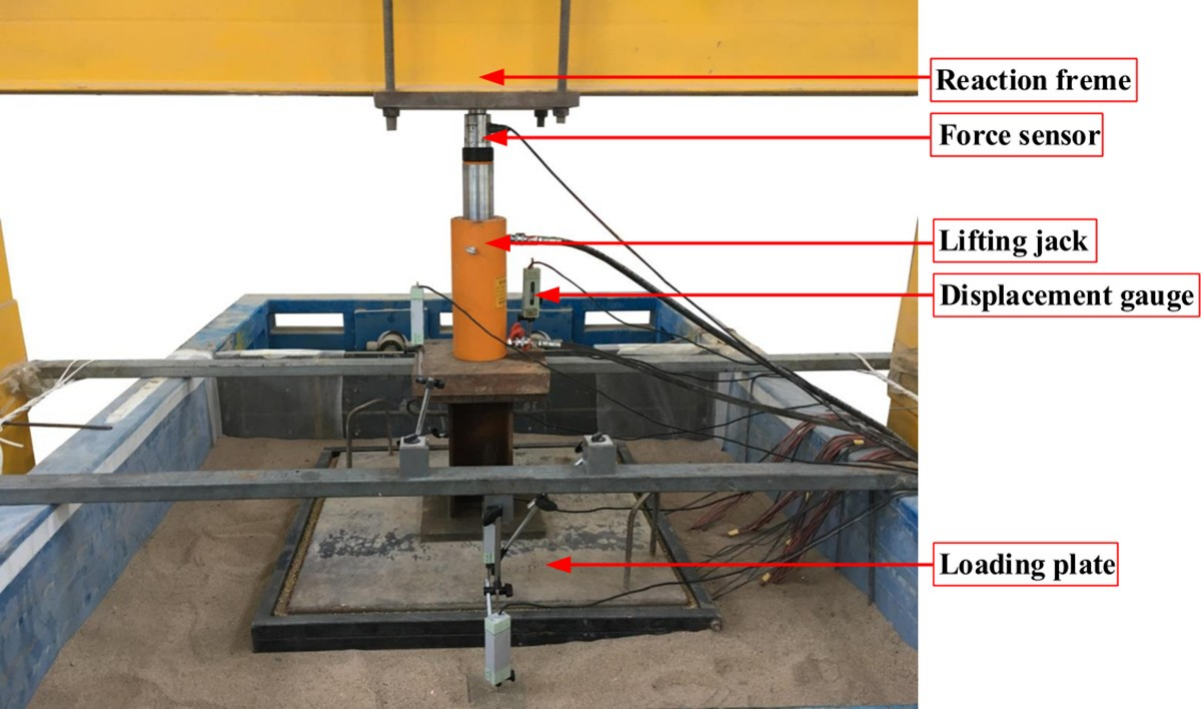
Photograph of the layout of the loading plate and lifting jack.

Before installing the piles, the backfill was filled to the height of the bottom of the long pile, then the pile was installed in the model box, and planks and level bars were used to ensure the planimetric position and verticality of the pile (see Fig 12). The filling method strongly influences the bearing behavior of the foundation. Due to the density of the backfill filled by the air pluviation method is too small to meet the test requirements, in this experiment, it is necessary to ensure that the density of the backfill is 1611 kg/m^3^.Therefore, the backfill of 891.6 kg is weighed first, and the air pluviation method is used to fill the box to make the soil distribution uniform. The soil is then compacted with a 10 kg hammer until the height of the backfill of this layer reaches 30 cm. In this way, the backfill is filled layer by layer to the height of the top of the piles. Fig 13 shows a photograph of the layout of the loading plate and lifting jack.

**Fig 14:**
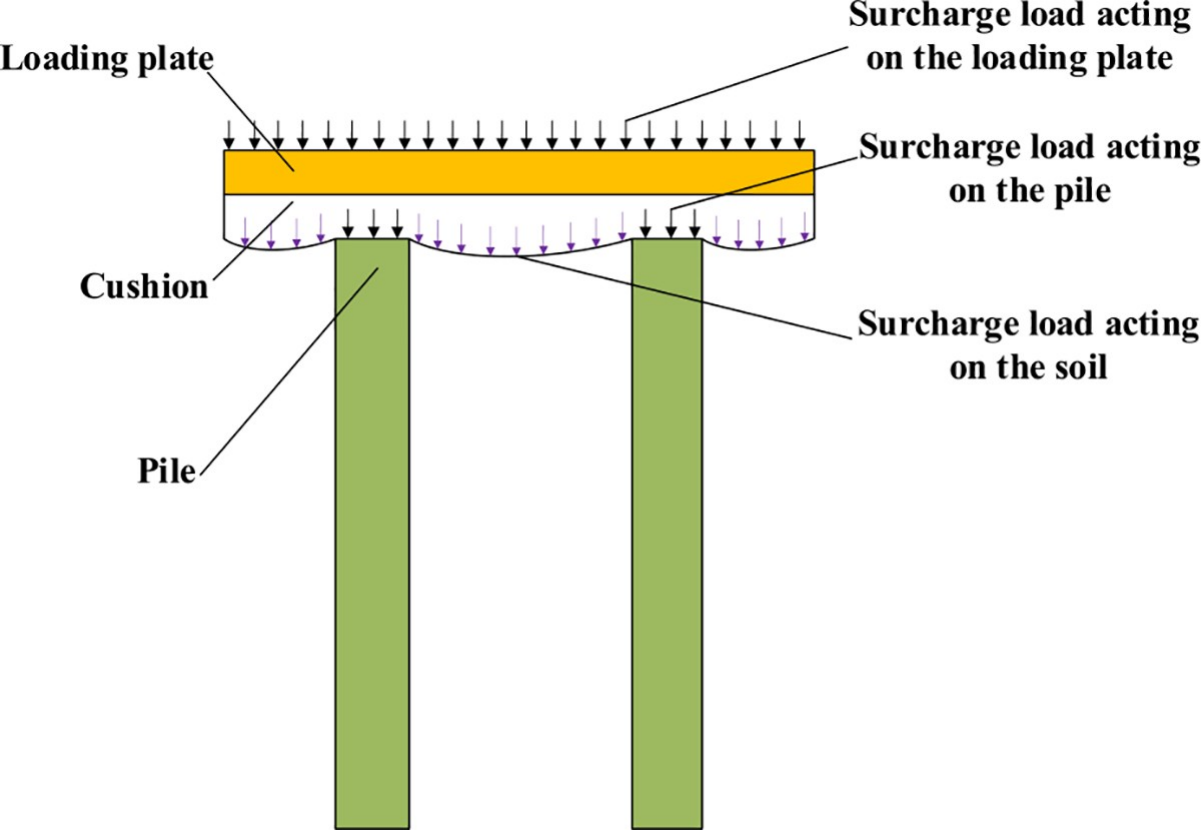
Schematic diagram of the pile-soil load-sharing principle of composite foundation.

Under the loading condition, a surcharge load was applied to the foundation. When *s/b =* 0.01 (where *s* is the settlement of the loading plate and *b* is the width of the loading plate), the surcharge load acting on the NG is determined as the final load of the NG. Fig 14 shows that in the composite foundation, the surcharge load acting on the loading plate is divided into two parts, in which one is the surcharge load acting on the pile and the other is the surcharge load acting on the soil. To study the influence of the surcharge load transmitted by the pile on the lateral earth pressure of the adjacent retaining structure, we kept the surcharge load acting on the soil in the CFRLP equal to that of the NG. Therefore, when the surcharge load acting on the soil between the piles of the CFRLP was equal to the final load of the NG, the surcharge load acting on the loading plate of the CFRLP was the final load of the CFRLP.

**Fig 15:**
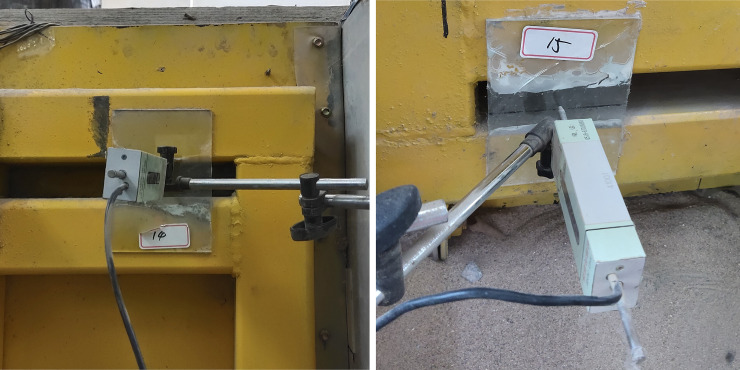
Displacement gauge for measuring the displacement of the movable rigid retaining structure. (a) Displacement gauge for measuring the top displacement of the retaining structure. (b) Displacement gauge for measuring the displacement of the bottom of the retaining structure.

In the rotation condition, the surcharge load was maintained, and the rigid retaining structure was rotated through 10 stages. The displacement of the top of the retaining structure in each stage is 1 mm, and there is no displacement at the bottom of the retaining structure. Four displacement gauges are symmetrically arranged at the top and bottom of the movable retaining structure to measure the displacement of the retaining structure in real time. A photograph of the displacement gauge is shown in Fig 15. The test was terminated when the displacement at the top of the retaining structure reached 10 mm.

Two groups of tests were carried out. The objective of Test 1 was to determine the earth pressure acting on the retaining structure adjacent to the NG, and the objective of Test 2 was to ascertain the earth pressure acting on the retaining structure adjacent to the CFRLP.

In the model test, data were collected for the lateral earth pressure acting on the retaining structure, the earth pressure between the piles, and the settlement of the foundation.

## 3. Results and discussion

This section presents and discusses the stress distribution, the additional earth pressure and the location of the total thrust on the retaining structure due to the surcharge on the foundations and rotation of the retaining structure in both NG and CFRLP.

### 3.1 Applying surcharge to the foundations

#### 3.1.1 Load-settlement curves

**Fig 16:**
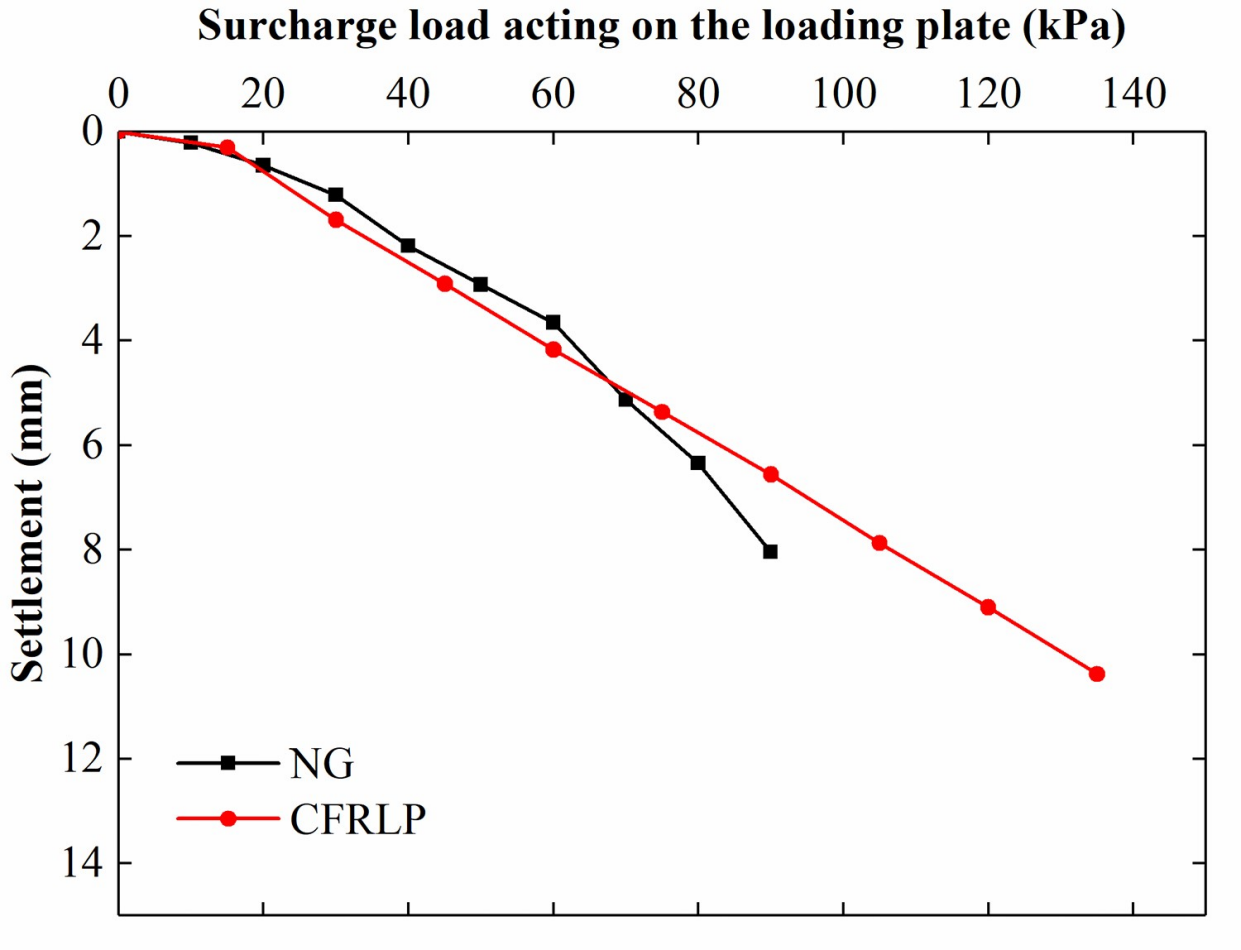
Load-settlement curves for the NG and CFRLP.

The load-settlement curves for the NG and CFRLP are shown in Fig 16. The characteristic value of the bearing capacity [32] for the NG was 89 kPa.

To study the influence of the surcharge load transmitted by the pile on the lateral earth pressure of the adjacent retaining structure, we kept the surcharge load acting on the soil in the CFRLP equal to that acting on the soil of the NG. The influence of the pile on the lateral earth pressure of the adjacent retaining structure was analyzed by comparing the results of the CFRLP and NG. Table 2 shows the surcharge load acting on the loading plate of the NG and CFRLP. When the surcharge loads acting on the loading plates in the NG and CFRLP were 89 kPa and 135 kPa, respectively, the surcharge load acting on the soil of both foundations was 89 kPa.

**Table 2:** The surcharge load acting on the loading plate of the NG and CFRLP.

Surcharge load acting on the NG (kPa)	10	20	30	40	50	60	70	80	89
Surcharge load acting on the loading plate of the CFRLP (kPa)	18.8	38.8	48.4	63.2	78.8	94.4	107.7	120.8	135.0

#### 3.1.2 The pile-soil stress ratio

**Fig 17:**
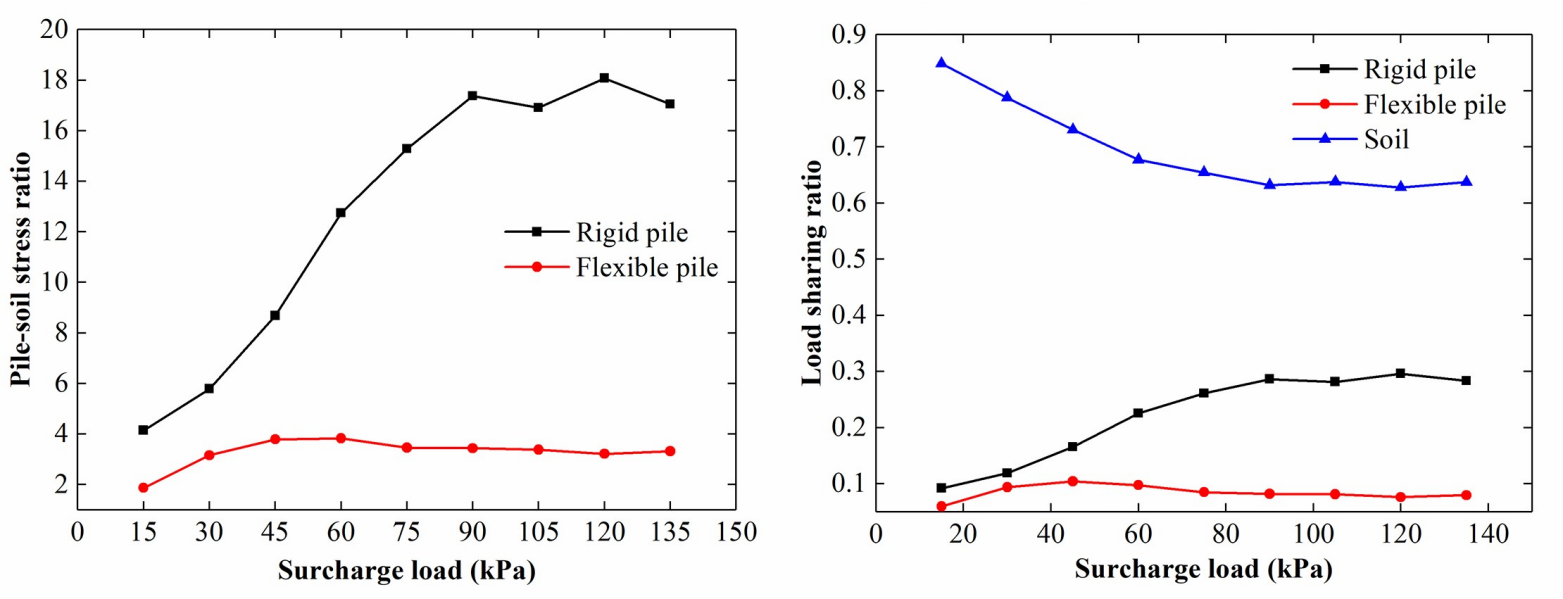
Pile-soil stress ratio and load-sharing ratio of the CFRLP due to applying surcharge. (a) Pile-soil stress ratio for different surcharge loads. (b) Load-sharing ratio for different surcharge loads.

The pile-soil stress ratio was calculated as *n = S*_*p*_ */ S*_*s*_ (where *S*_*p*_ is the axial stress of the pile and *S*_*s*_ is the vertical stress of the soil). The load-sharing ratio of the pile was calculated as *λ*_*p*_ *= L*_*p*_ */ L* (where *L*_*p*_ is the surcharge load acting on the pile, and *L* is the surcharge load acting on the loading plate). The load-sharing ratio of the soil was calculated as *λ*_*s*_ *= L*_*s*_ */ L* (where *L*_*s*_ is the surcharge load acting on the soil between the piles, and *L* is the surcharge load acting on the loading plate). As shown in Fig 17, the pile-soil stress ratio of the rigid pile changed significantly during the loading process. The pile-soil stress ratio of the rigid pile increased from 5 to 17 and stabilized at approximately 17. The load-sharing ratio of the rigid pile increased from 0.1 to 0.28 and stabilized at approximately 0.28. The change in the pile-soil stress ratio and the load-sharing ratio of the flexible pile was relatively small; the former fluctuated at approximately 3, and the latter fluctuated at approximately 0.8.

#### 3.1.3 The additional earth pressure

**Fig 18:**
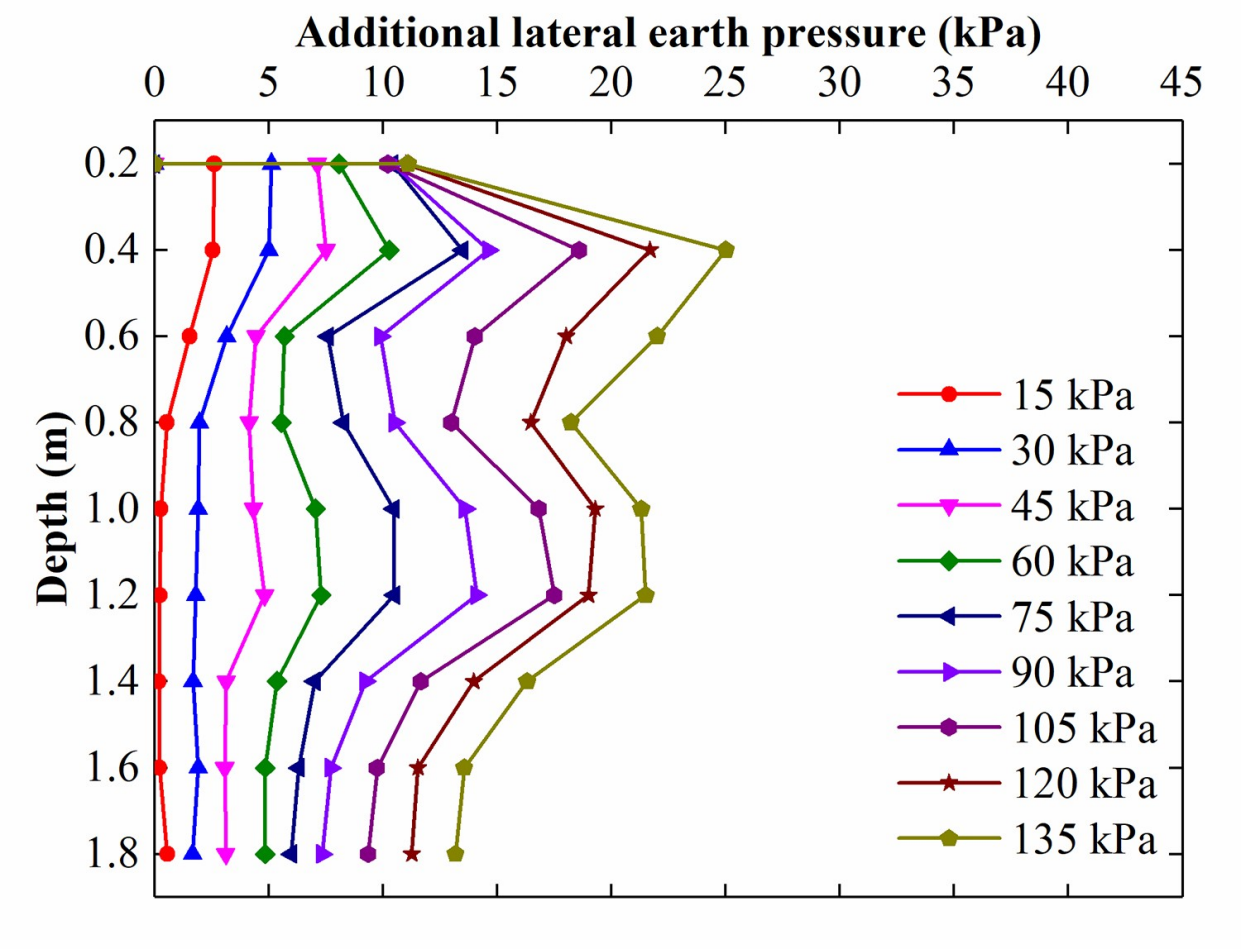
The additional lateral earth pressure acting on the retaining structure adjacent to the CFRLP for different surcharge loads.

Fig 18 illustrates the additional lateral earth pressure acting on the retaining structure in the soil adjacent to the CFRLP for different surcharge loads. At a surcharge of 15 kPa, the additional earth pressure had a maximum value at a depth of 0.2 m, decreased with increasing depth, and became negligible once the depth was greater than 0.8 m (approximately the width of the foundation). This is because when the loading was small, the load-sharing ratio of the rigid piles was less than 10%, as illustrated in Fig 17, and most of the load was carried by the soil and transferred to the retaining structure directly. As the surcharge load increased, the magnitude of the additional lateral earth pressure increased in the range of 0.4 m-1 m. This is because with the increase in surcharge, the pile-soil stress ratio increased, which means that greater stress was carried by the piles and transferred into deeper zones.

**Fig 19:**
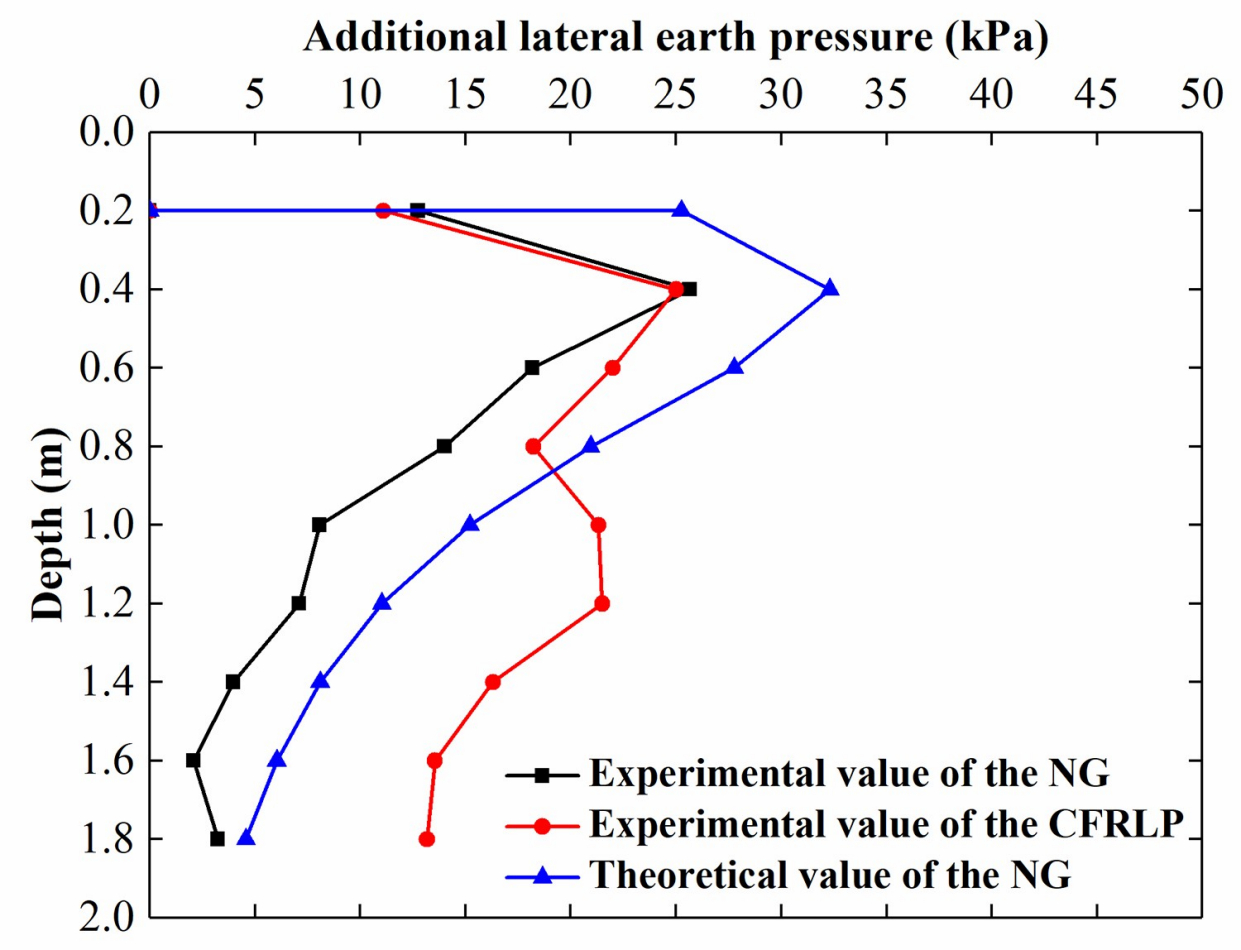
Additional lateral earth pressure for a surcharge load of 89 kPa acting on the soil.

Fig 19 shows the additional lateral earth pressure acting on the retaining structure when the stress acting on the soil was 89 kPa in both the NG and the CFRLP. The theoretical method [33–35] derived from the Boussinesq solution was used to calculate the additional lateral earth pressure acting on the retaining structure adjacent to the NG, as shown in Fig 19.

As indicated in Fig 19, the trend in the theoretical results for the NG is comparable to that of the experimental results. The magnitude of the additional lateral earth pressure acting on the retaining structure increased sharply with increasing depth between 0.2 m and 1 m below the ground and then decreased abruptly along the depth. Below a depth of 1 m, the additional lateral earth pressure acting on the retaining structure was comparatively small.

In addition, unlike in NG, where the external load primarily affected the additional lateral earth pressure in the shallow area, in the CFRLP, the magnitude of the lateral earth pressure acting on the retaining structure was almost the same as that in NG between 0.2 m and 0.6 m below the ground, but much greater beyond 0.8 m. This may be attributed to the fact that the stiffness of the flexible pile was similar to that of the soil, and the flexible pile and soil acted as a reinforcement area, which led to the flexible pile improving the bearing capacity of the reinforced area and ensuring that the reinforced area could transfer the surcharge load to the deep soil. As the area reinforced by the flexible piles transferred the surcharge load to the deeper soil, the lateral earth pressure acting on the retaining structure adjacent to the CFRLP was greater than that adjacent to the NG between 0.6 m and 1.2 m below the ground.

**Fig 20:**
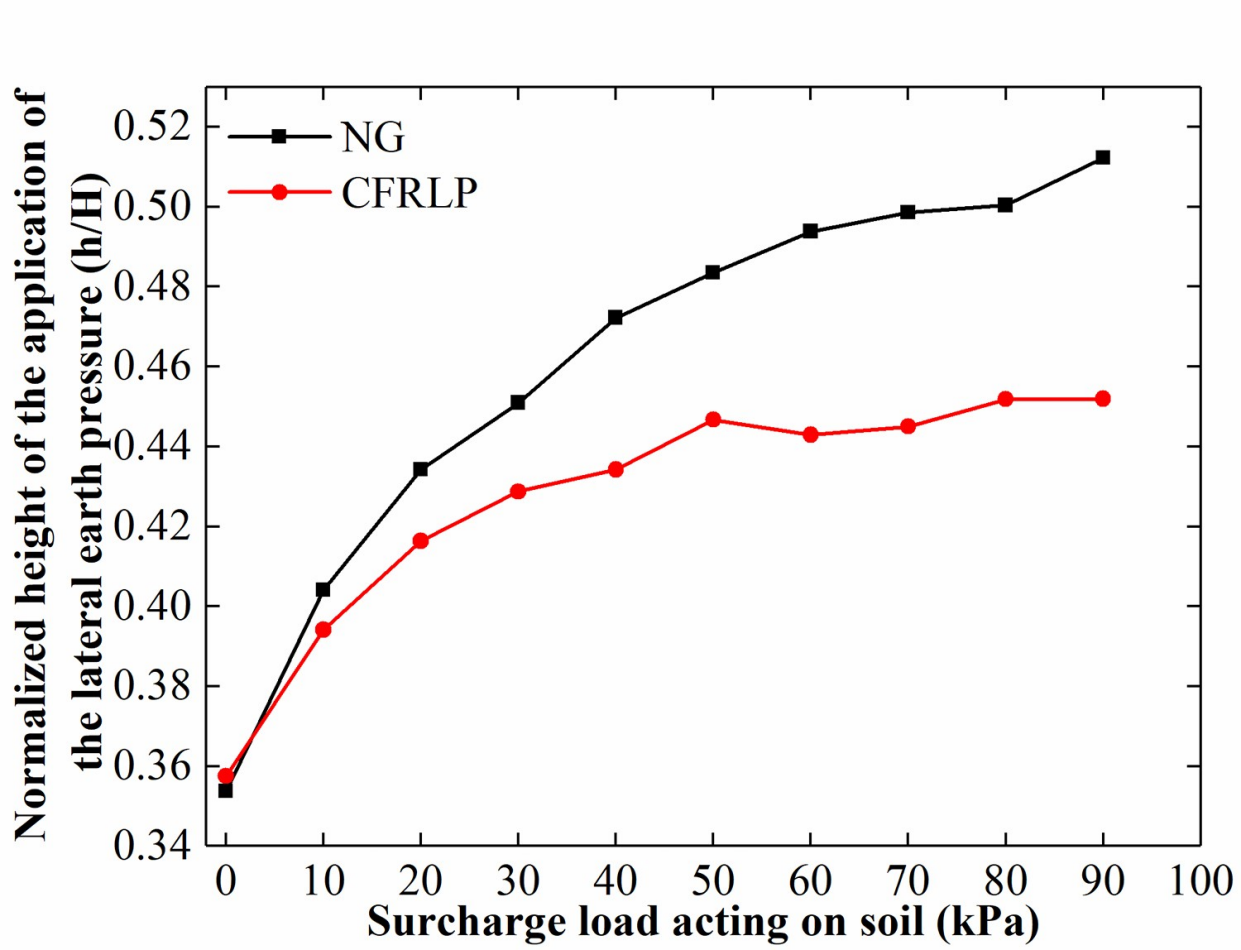
The normalized height of the application of the lateral earth pressure for different surcharge loads.

The height of application of the lateral earth pressure is an important indicator for evaluating the stability of the retaining structure in excavation engineering applications. Fig 20 shows that the normalized height of application of the lateral earth pressure acting on the retaining structure without a load is 0.35 H above the bottom of the retaining structure (H is the height of the retaining structure). The normalized height of application of the lateral earth pressure acting on the retaining structure adjacent to the NG increases steadily with an increase in the surcharge load. In contrast, the normalized height in the CFRLP increases at a lower rate than that of the NG and stabilizes at approximately 0.44 H.

Such a phenomenon may also be attributed to the inability of the NG to transfer the surcharge load to the deep soil, resulting in the height of application of the lateral earth pressure increasing with an increase in the surcharge load. In contrast, once the surcharge load reaches 55% of the final surcharge load, the load-sharing ratio of the reinforced area and the rigid pile of the CFRLP remains unchanged with an increase in the surcharge load; therefore, both the proportion of the load transferred from the CFRLP to the upper part of the retaining structure and the proportion transferred to the lower part remain unchanged. Therefore, the height of application of the lateral earth pressure acting on the retaining structure adjacent to the CFRLP stabilizes.

### 3.2 Rotating the retaining structure

#### 3.2.1 The pile-soil stress ratio

**Fig 21:**
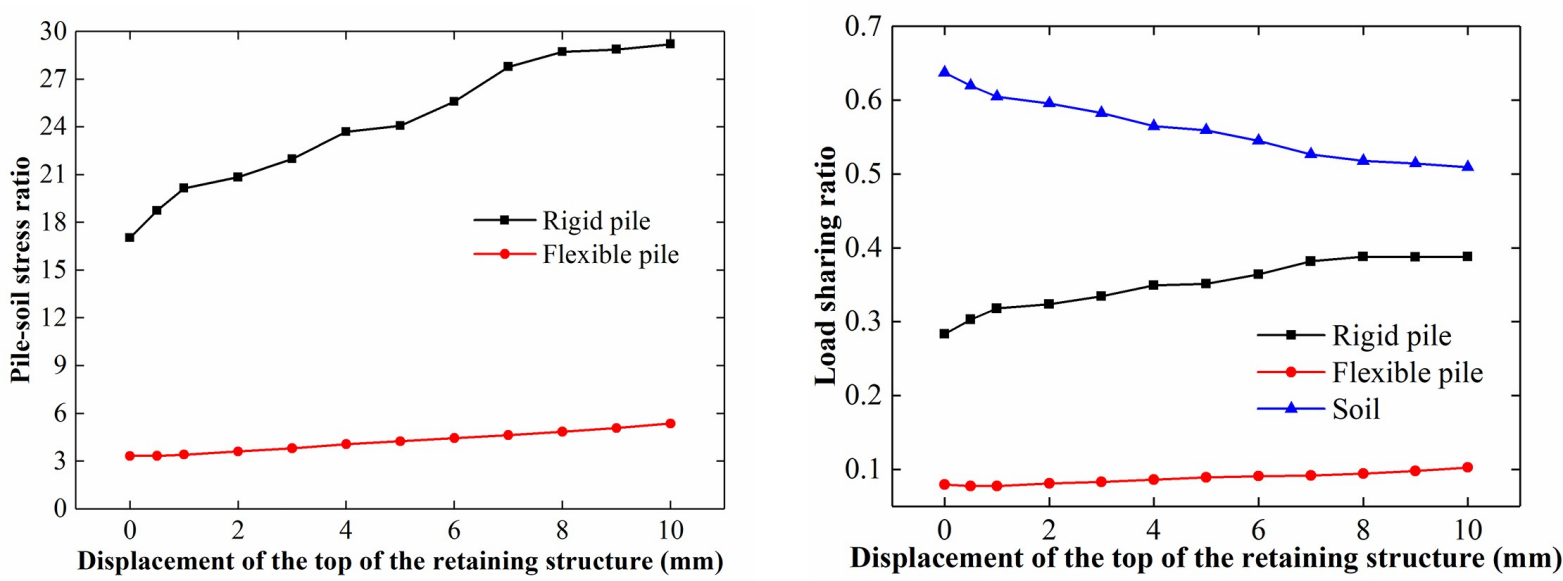
Pile-soil stress ratio and load-sharing ratio of the CFRLP due to the rotation of the retaining structure. (a) Pile-soil stress ratio for different displacements of the top of the retaining structure. (b) Load-sharing ratio for different displacements of the top of the retaining structure.

When rotating the retaining structure along its toe, as shown in Fig 21, the pile-soil stress ratio and load-sharing ratio of the pile increased with an increase in the rotation of the retaining structure, and the changes were more significant for the rigid pile than for the flexible pile. The lateral constraint of the soil between the piles decreased with increasing rotation of the retaining structure, decreasing the vertical bearing capacity of the soil between the piles. In addition, the settlement of the soil between the piles increased with a decrease in the vertical bearing capacity of the soil between the piles. Due to increased settlement, the surcharge load borne by the soil between the piles decreased, resulting in an increase in the pile-soil stress ratio and load-sharing ratio of the piles to varying degrees.

#### 3.2.2 The additional lateral earth pressure

**Fig 22:**
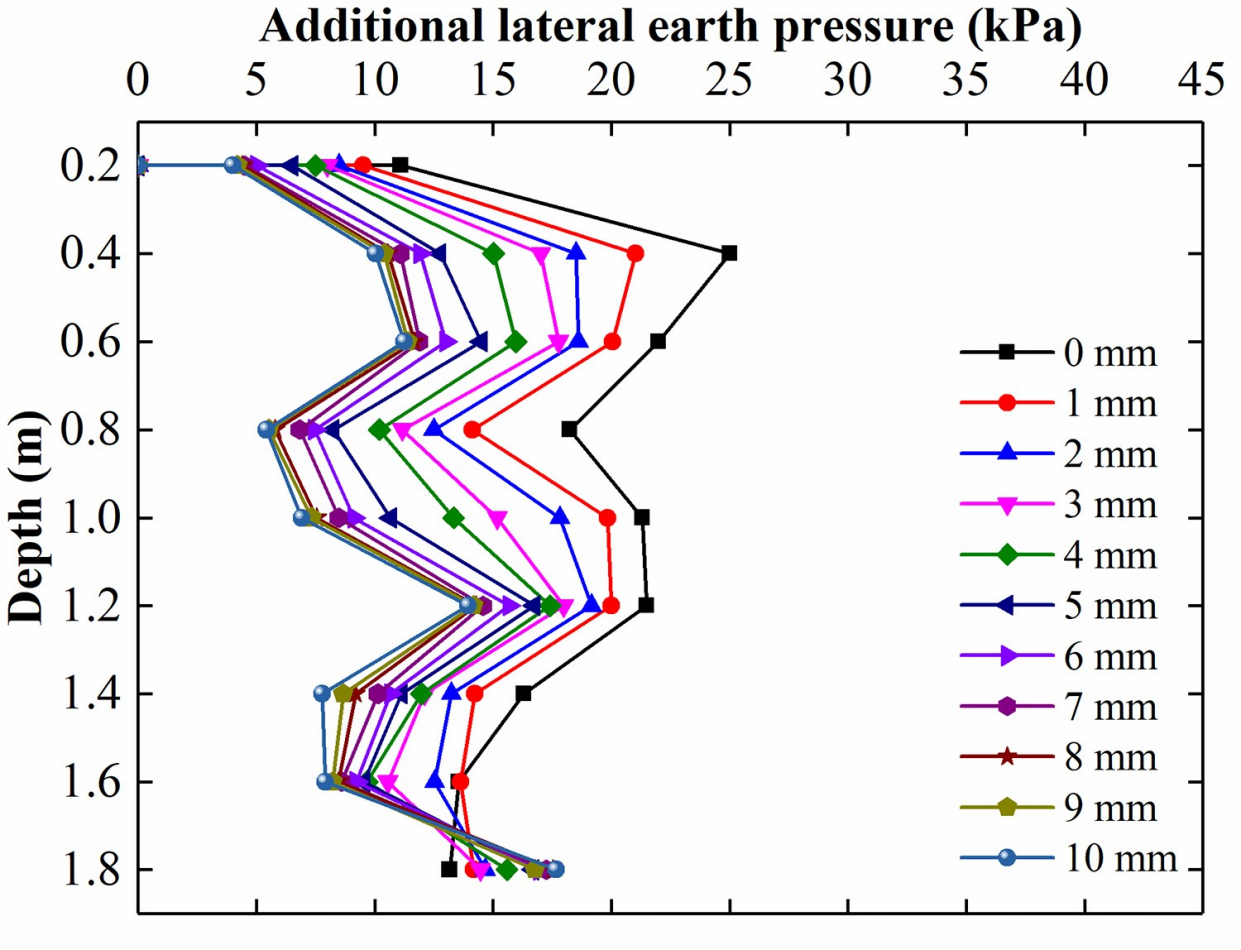
The additional lateral earth pressure adjacent to the CFRLP for different displacements of the top of the retaining structure.

The additional lateral earth pressure adjacent to the CFRLP for different displacements of the top of the retaining structure is shown in Fig 22. The additional lateral earth pressure acting on the retaining structure above a depth of 1.6 m decreased with increasing rotation of the retaining structure. This is because the load-sharing ratio of the soil decreased with an increase in the rotation of the retaining structure, as shown in Fig 21, resulting in the magnitude of the lateral earth pressure transferred from the soil to the retaining structure decreasing. In addition, during rotation, the horizontal displacement of the upper part of the retaining structure was relatively large, and the internal shearing resistance of the soil between the CFRLP and retaining structure was mobilized, resulting in a decrease in the stress diffusion ability of the soil. As a result of the two factors, the magnitude of the additional lateral earth pressure of the retaining structure at a depth above 1.6 m decreased with an increase in the rotation of the retaining structure.

For depths below 1.6 m, the additional lateral earth pressure acting on the retaining structure increased with increasing rotation of the retaining structure. This is because the surcharge load transferred from the piles to the deep soil increased with an increase in the rotation of the retaining structure (see Fig 21), increasing the stress in the deep soil. In addition, the displacement of the lower part of the retaining structure barely changed with the rotation of the retaining structure. Therefore, the magnitude of the additional lateral earth pressure acting on the lower part of the retaining structure increased with an increase in the rotation of the retaining structure.

**Fig 23:**
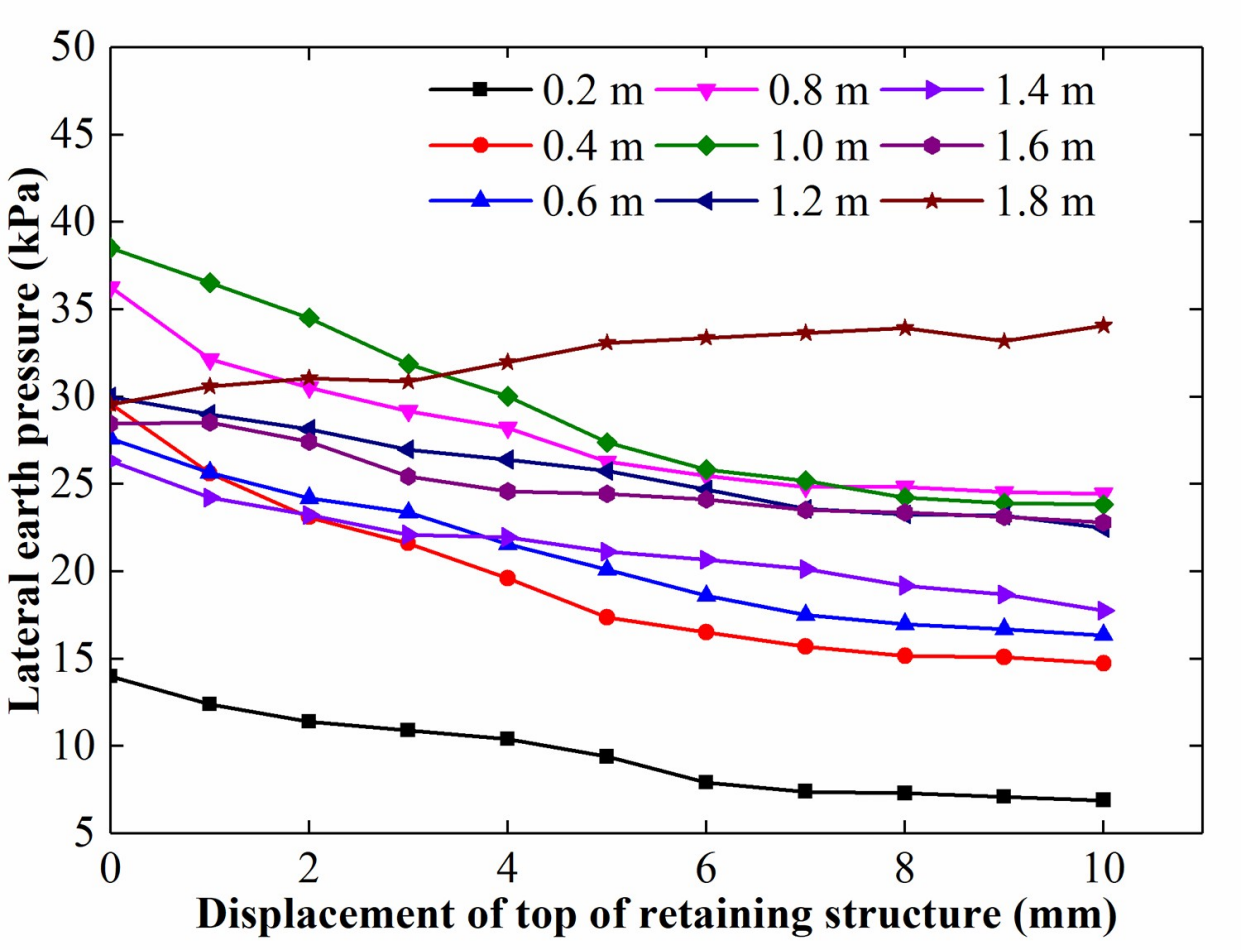
The lateral earth pressure at different depths for different displacements of the retaining structure.

The lateral earth pressure at different depths for different displacements of the retaining structure is shown in Fig 23. The rate of change in the lateral earth pressure was almost zero for a depth above 1.2 m when the displacement of the top of the retaining structure reached 8 mm (0.04% H).

**Fig 24:**
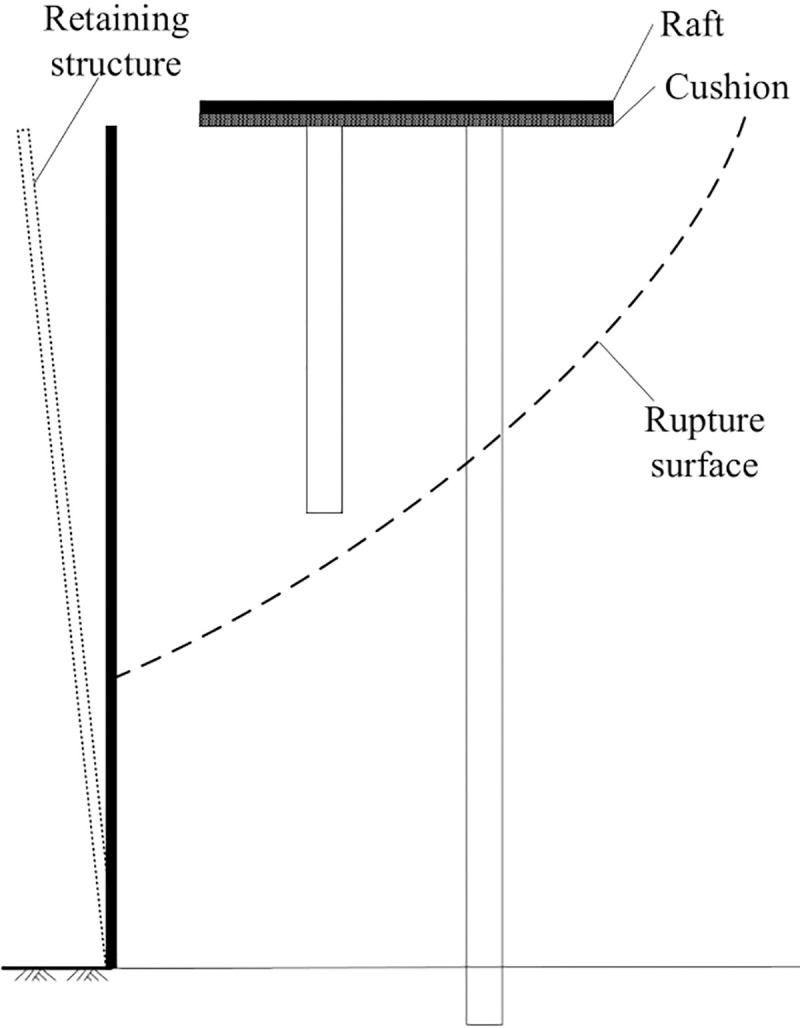
Schematic diagram of the rupture surface.

The abovementioned phenomenon may occur because the soil behind the rotating structure reached an active limiting state. In the limiting state, a sliding wedge develops in the soil behind the rotating structure (see Fig 24). The soil slides horizontally along an inclined plane separating the wedge from the remaining soil mass, and the internal shearing resistance of the sliding wedge is fully mobilized. This results in further surcharge load not being transferred to the retaining structure. In contrast, the displacement of the lower part of the retaining structure is smaller than that of the upper part, suggesting that the deep soil may not reach the active limiting state. Therefore, the magnitude of the lateral earth pressure acting on the lower part of the retaining structure continues to change with the rotation of the retaining structure.

**Fig 25:**
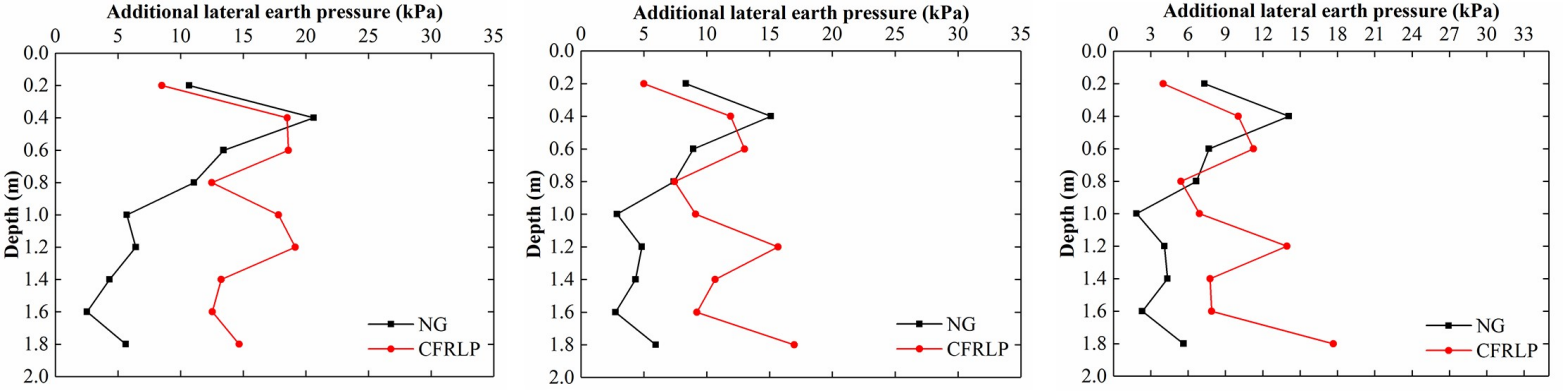
The additional lateral earth pressure values for different displacements of the top of the retaining structure. (a) 2 mm. (b) 6 mm. (c) 10 mm.

As shown in Fig 25, the reduction in the additional lateral earth pressure acting on the retaining structure was larger for the area adjacent to the CFRLP than for that adjacent to the NG between 0 m and 0.6 m below the ground. Such a trend may be attributed to the load-sharing ratio of the soil decreasing with an increase in the rotation of the retaining structure (see Fig 21), resulting in the additional stress transferred from the soil to the retaining structure decreasing. In addition, the shielding effect of the flexible pile weakened the ability of the soil to transfer the surcharge load to the retaining structure. Furthermore, since the ability of the NG to transfer the surcharge load to the deep soil was weak, most of the load was concentrated in the shallow area of the soil, which resulted in the additional stress in the shallow area of the NG being much larger than that of the CFRLP in the rotation test.

**Fig 26:**
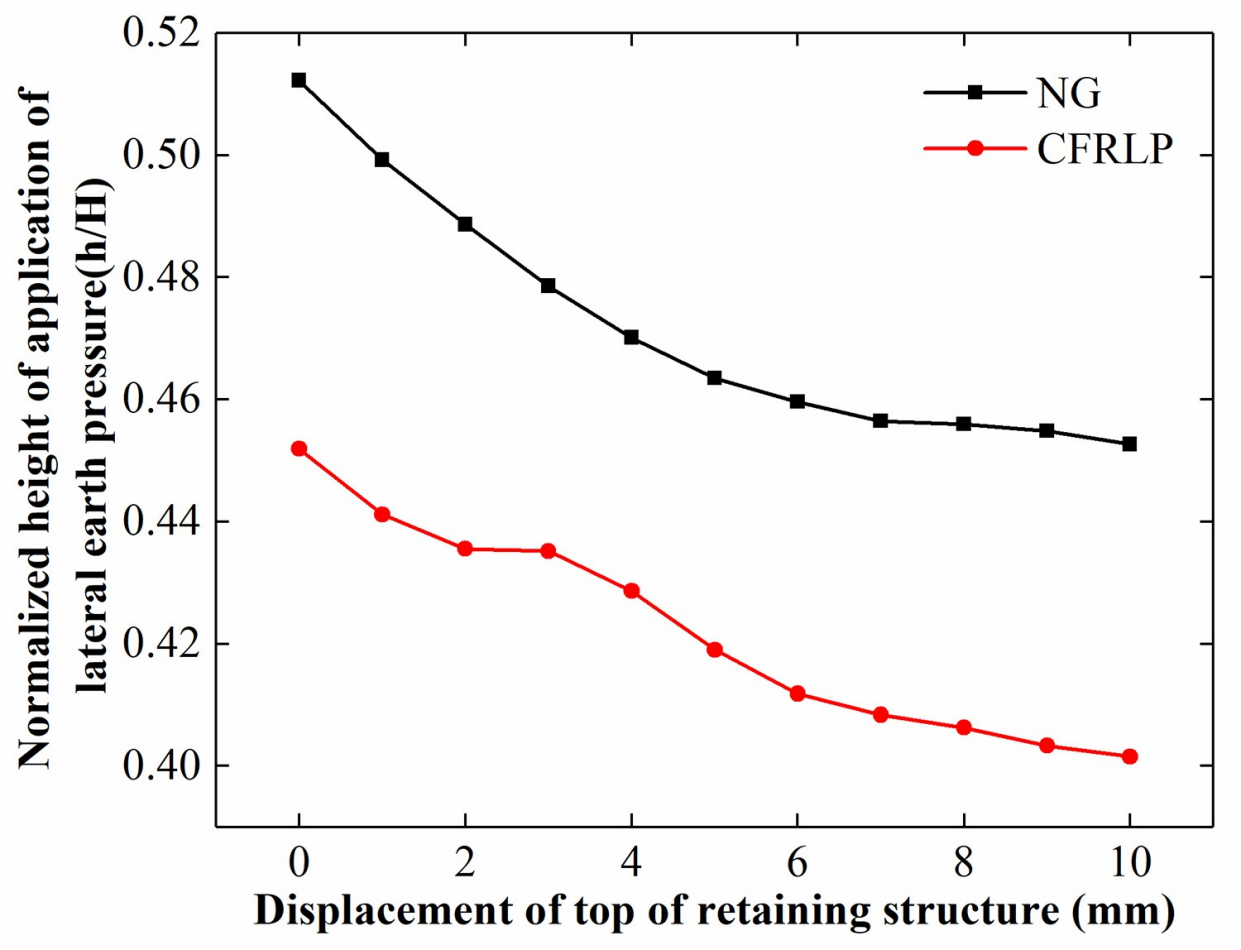
The normalized height of application of the lateral earth pressure for different displacements of the top of the retaining structure.

Fig 26 shows that the height of application of the lateral earth pressure of the NG and CFRLP decreased with an increase in the rotation of the retaining structure. This may be because the upper soil entered the active state earlier than the lower soil as the rotation of the retaining structure increased, resulting in a greater reduction in the lateral earth pressure in the upper part. Since the surcharge load to the deep soil increased with an increase in the rotation of the retaining structure, the lateral earth pressure acting on the lower part of the retaining structure increased with the rotation of the retaining structure.

## 4. FEM analysis

In the previous section, the distribution and evolution mechanism of the lateral earth pressure acting on the retaining structure adjacent to the CFRLP were described in detail through a model test. In this section, a three-dimensional FEM was used to analyze the lateral earth pressure, and the effect of the parameters of the flexible pile were investigated.

### 4.1 FEM model

**Fig 27:**
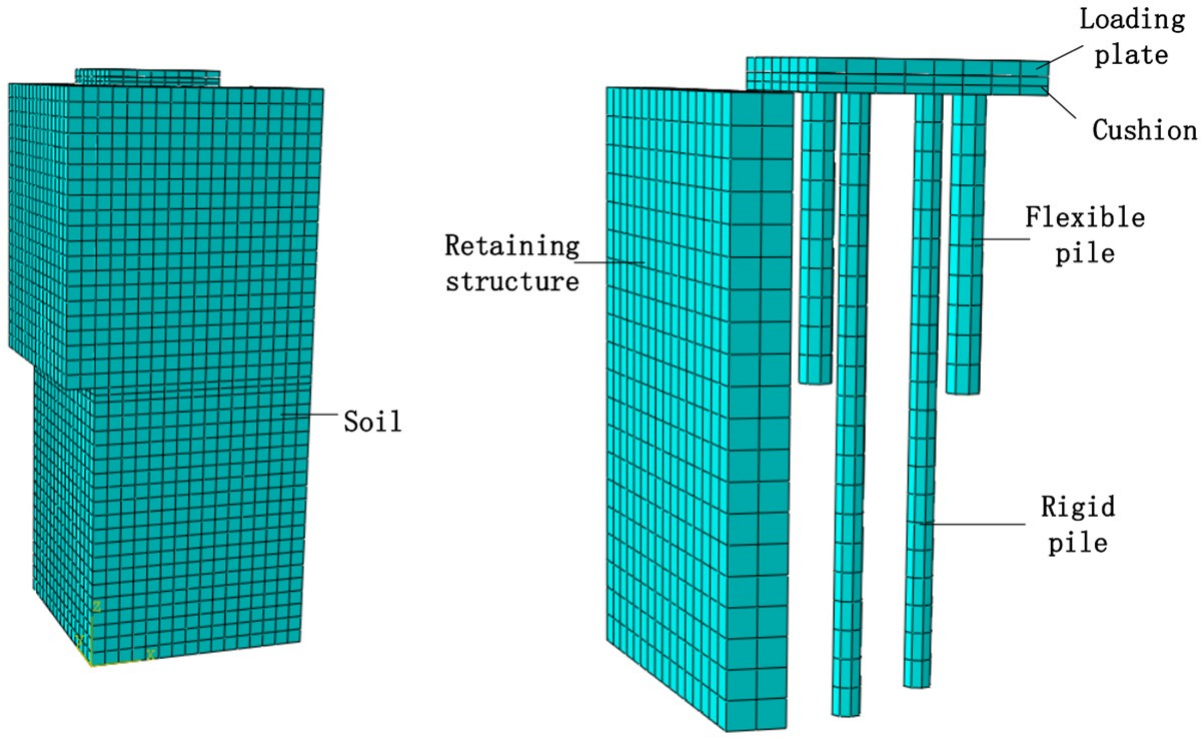
Arrangement of the numerical model.

The size of each component in the FEM analysis is equal to that of the model test. The length of the long pile is 2.1 m, and the diameter is 100 mm. The length of the flexible pile is 1 m, and the diameter is 120 mm. The size of the backfill is 1.6 m (length) × 1.6 m (width) × 4 m (height). The size of the cushion is 800 mm (length) × 800 mm (width) × 60 mm (height). The arrangement of the numerical model is shown in Fig 27.

The backfill and the cushion in the FEM analysis were modeled as elastic-plastic materials following a Mohr-Coulomb criterion, while the piles and retaining structure were assumed to be linearly elastic materials. The material properties of the various components are shown in Table 3.

**Table 3:** Material properties used in the FEM analysis.

Material	ρ (kg/m^3^)	E (MPa)	υ	c (kPa)	φ (°)
Backfill	1611	20	0.3	0.2	33.42
Cushion	1400	30	0.29	5	33.9
Rigid pile	2500	13000	0.2	-	-
Flexible pile	2500	400	0.2	-	-
Loading plate	7800	210000	0.2	-	-
Retaining structure	7800	210000	0.2	-	-

Under the loading condition, the bottom boundary of the FEM model was fixed in three directions and the lateral surrounding boundary was treated as vertically sliding but horizontally restrained. Under the rotation condition, the boundary restrictions near the side of the rigid movable retaining structure were canceled. The displacement of the bottom of the rigid movable retaining structure was kept unchanged, and the rotation of the retaining structure was then controlled by changing the displacement of the top of the rigid movable retaining structure. An 8-node linear brick mesh with reduced integration and hourglass control was used for the FEM model, and a relatively fine mesh was used near the pile-soil interface. Coulomb friction was used to simulate the interface between the soil and the pile. The pile-soil interface friction coefficient was obtained by a direct shear test. The interface friction coefficients *μ*_1_ = 0.66 and *μ*_2_ = 0.51 were selected for a flexible pile and a rigid pile, respectively.

### 4.2 Comparison

**Fig 28:**
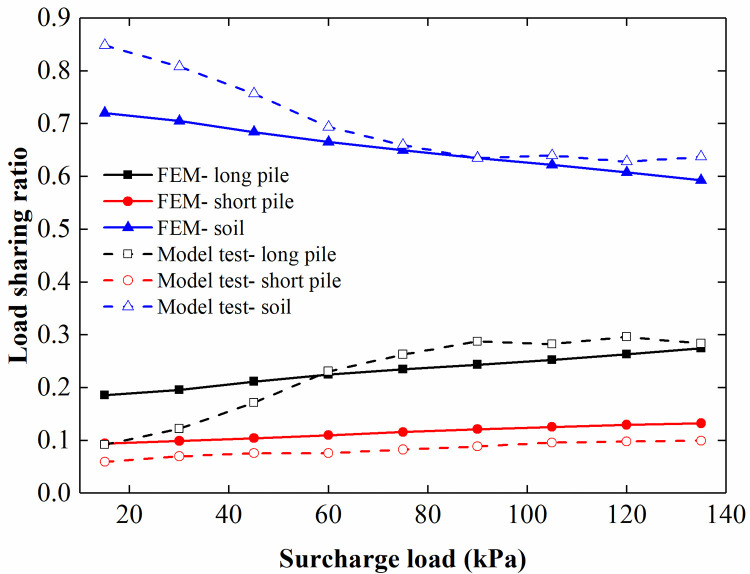
Comparison of the load-sharing ratio for different surcharge loads.

**Fig 29:**
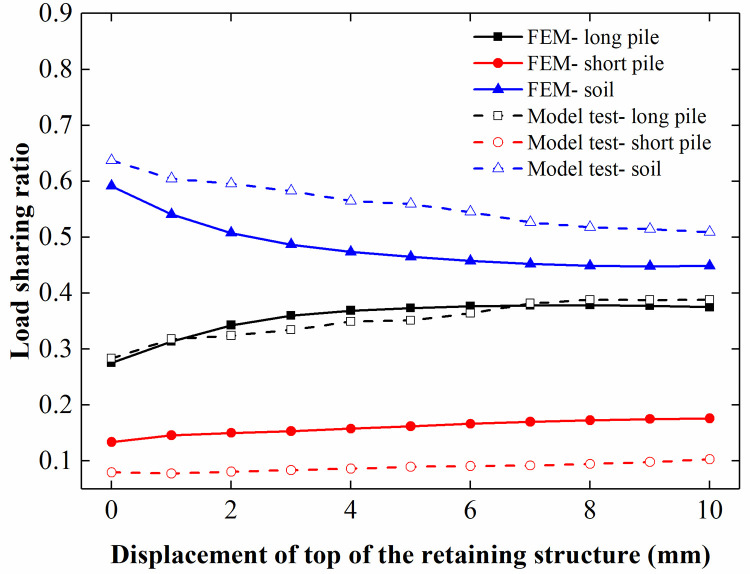
Comparison of the load-sharing ratio for different displacements of the retaining structure.

Fig 28 shows the comparison of the load-sharing ratios for different surcharge loads. In the FEM, the load-sharing ratio of the long pile increased with the increase in the surcharge load acting on the loading plate, the load-sharing ratio of the soil between piles decreased with the increasing surcharge load, and the load-sharing ratio of the short pile increased slightly with the increased surcharge load. In addition, Fig 29 shows that the load-sharing ratios of the long pile and short pile increased with the increasing rotation of the retaining structure, while the load-sharing ratio of the soil between piles decreased with the increased rotation of the retaining structure. After comparing the FEM analysis results with the model test results, we believe that the FEM results are similar to the model test results and that the change trend in the curve is also the same.

**Fig 30:**
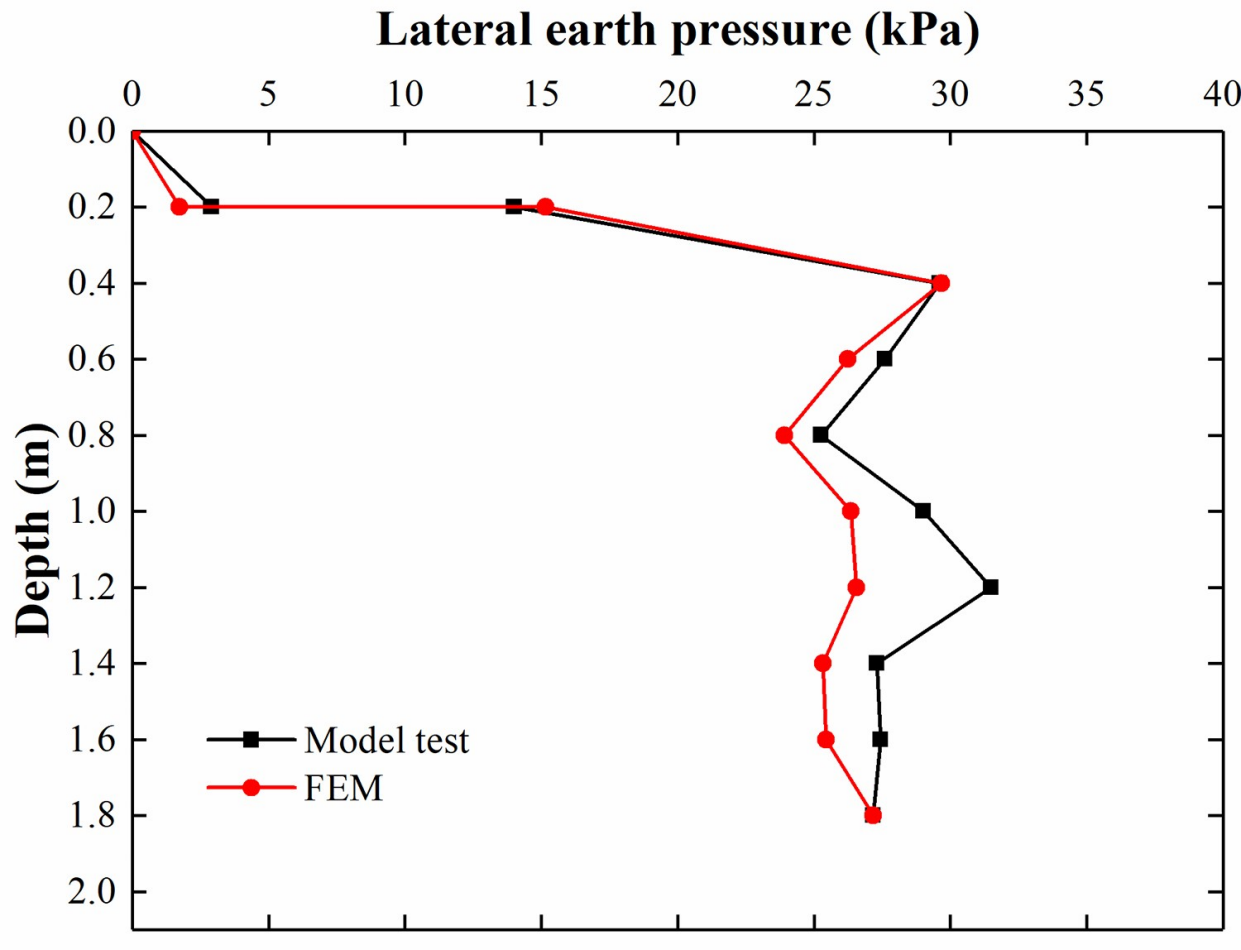
Comparison of the lateral earth pressure when the surcharge load is 135 kPa.

**Fig 31:**
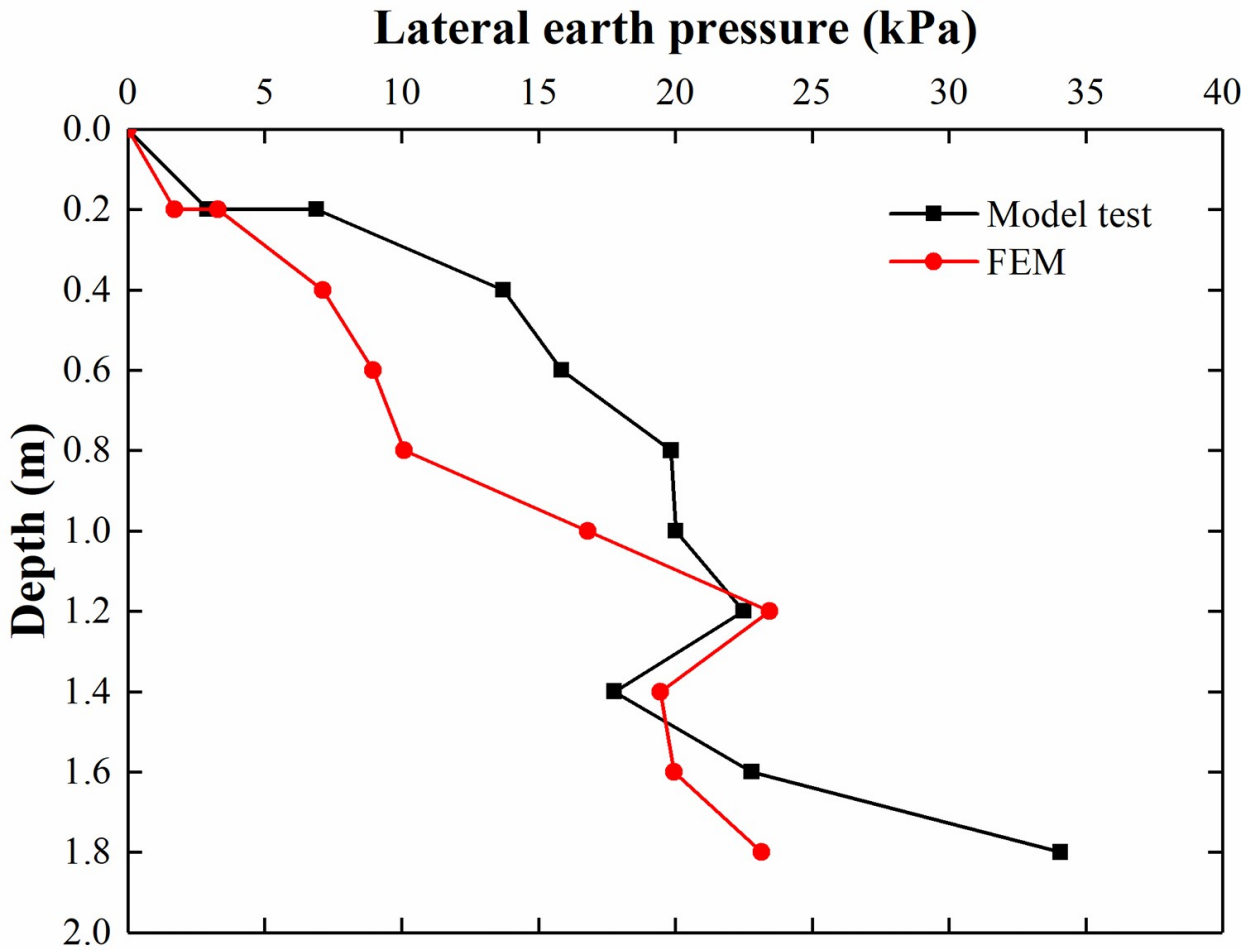
Comparison of the lateral earth pressure when the displacement of the retaining structure is 10 mm.

Fig 30 shows that the results of the FEM analysis are consistent with those of the model test at depths of 0.2–0.6 m. Because the model test conditions have difficulty achieving the ideal conditions of the FEM analysis, there are still some differences between the two results at depths of 0.8–1.8 m. As seen from Fig 31, the lateral earth pressure of the FEM analysis and model test increases with increasing depth and reaches the maximum value at 1.2 m. The trend of the FEM analysis is comparable to that of the model test.

From the above comparison, it can be concluded that the lateral earth pressure obtained from the model test is reasonable and reliable.

### 4.3 Parametric study

Scholars have studied the effects of the elastic modulus and length of piles on the properties of composite foundations [1, 9, 10], while no studies have focused on the lateral earth pressure acting on the retaining structure adjacent to CFRLPs. To study the effect of the elastic modulus and length of flexible piles on the lateral earth pressure acting on the retaining structure, the elastic modulus of the flexible pile varies from 400–1200 MPa, the length of the flexible pile varies from 1–1.4 m, and the other parameters are the same as those above. It should be noted that the surcharge load acting on the soil in each model was 89 kPa in the parametric study of the elastic modulus and the length of the pile.

**Fig 32:**
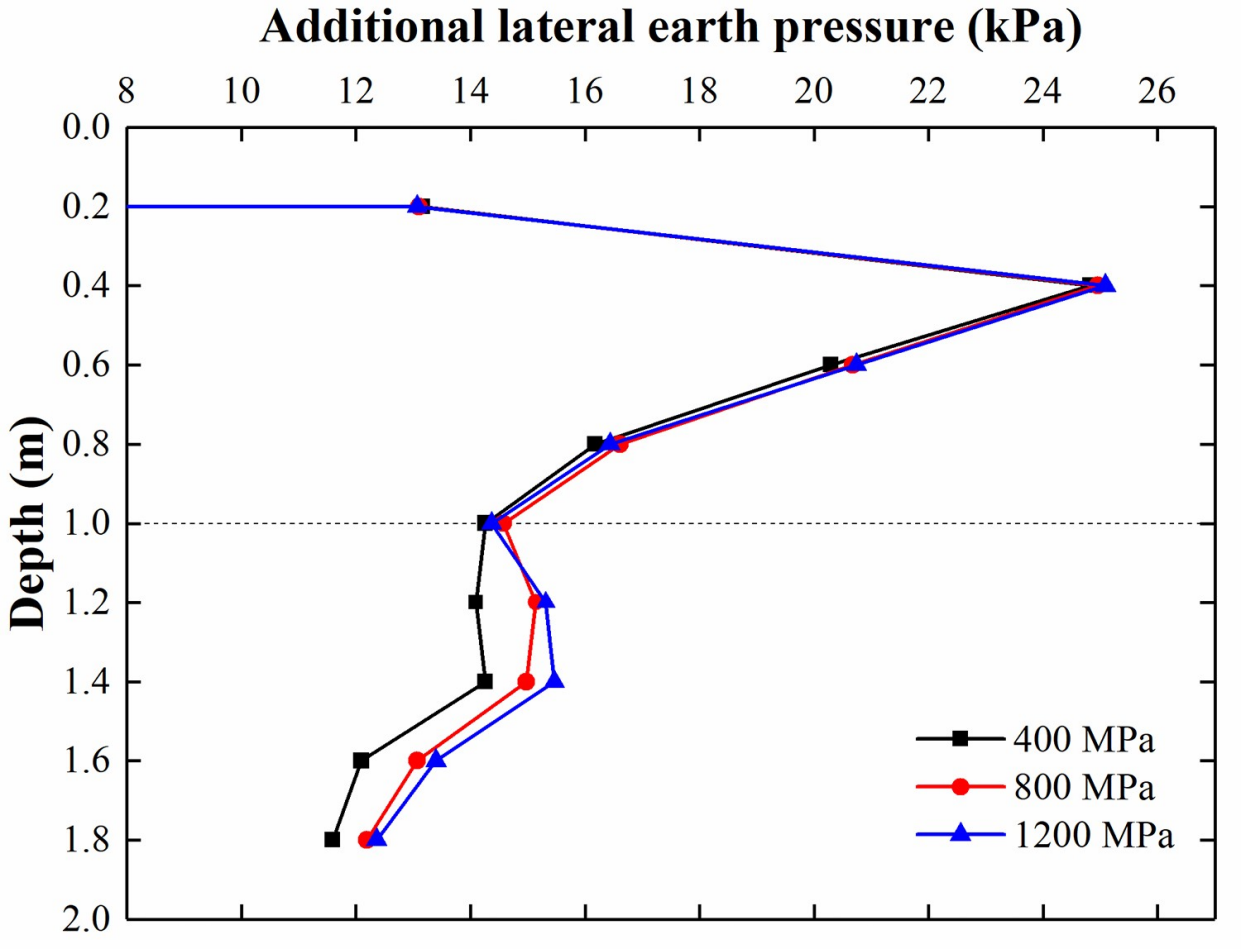
Comparison of the lateral earth pressure for different elastic modulus values for flexible piles.

**Fig 33:**
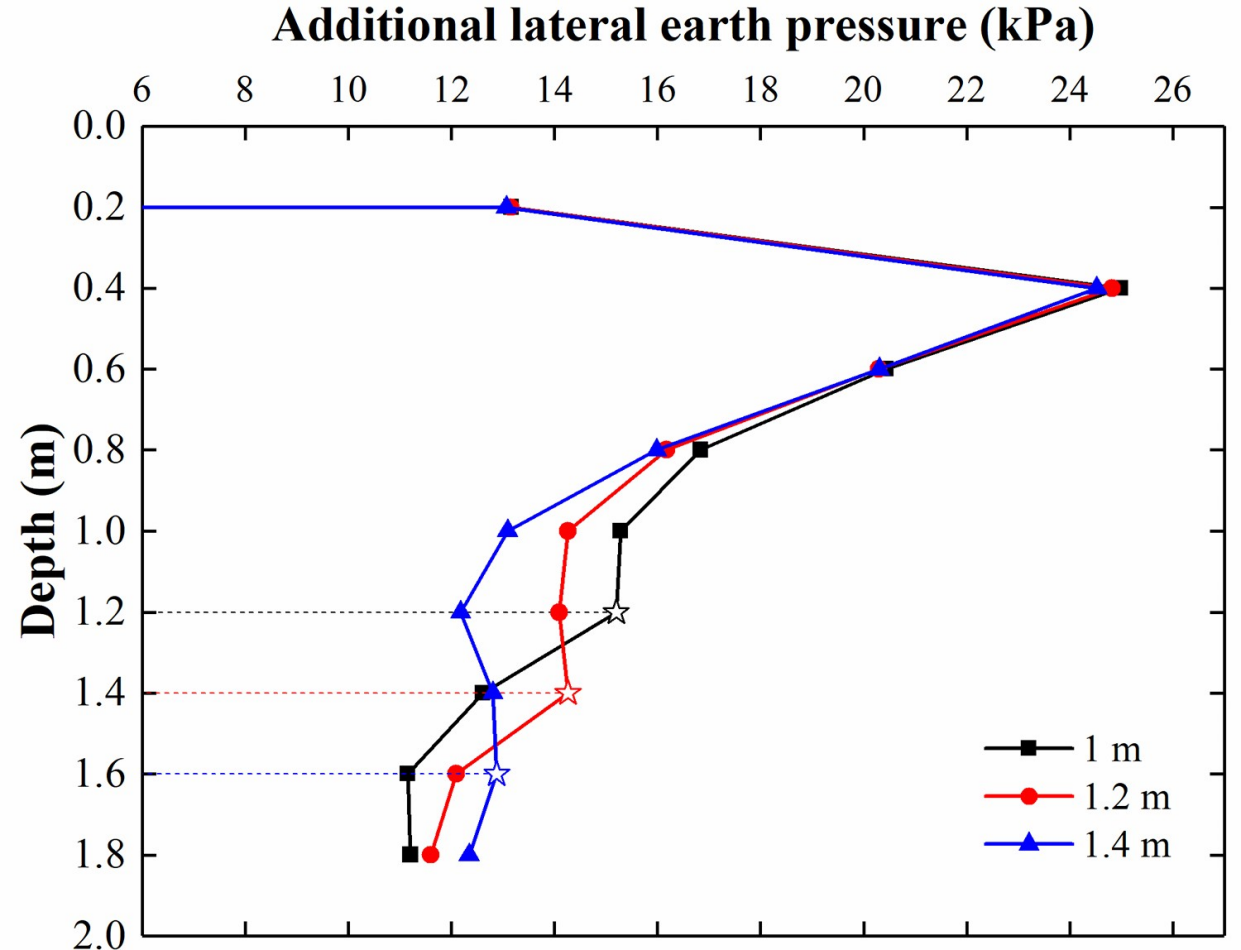
Comparison of the lateral earth pressure for different lengths of flexible piles.

Fig 32 illustrates the comparison of the lateral earth pressure with a varying elastic modulus of the flexible pile. It is clear that at a depth of 0–1 m, the additional lateral earth pressures are equal to one another in different FEM models with a varying elastic modulus of the flexible pile. This relationship exists because in the parametric study, the surcharge loads acting on the soil in different FEM models are equal to one another, and the surcharge loads borne by the soil and diffused to the adjacent retaining structure are also equal to one another. In addition, below a depth of 1 m, the lateral earth pressure reaches a maximum at 1.2 m, and the lateral earth pressure increases with increasing elastic modulus. This is because with the increase in the modulus of the flexible pile, the pile transfers more surcharge load to the subsoil, which leads to the increase in the lateral earth pressure acting on the retaining structure. Fig 33 shows the comparison of the lateral earth pressure for varying lengths of flexible piles. Below a depth of 1 m, the flexible piles of different lengths reach a maximum lateral earth pressure at different depths. The longer the pile is, the deeper the pile is embedded when the earth pressure reaches the maximum value. Such a trend can be attributed to the fact that as the length of the flexible pile increases, the position of the load transferred from the pile becomes deeper, which leads to the position of the maximum value of lateral earth pressure becoming deeper.

**Fig 34:**
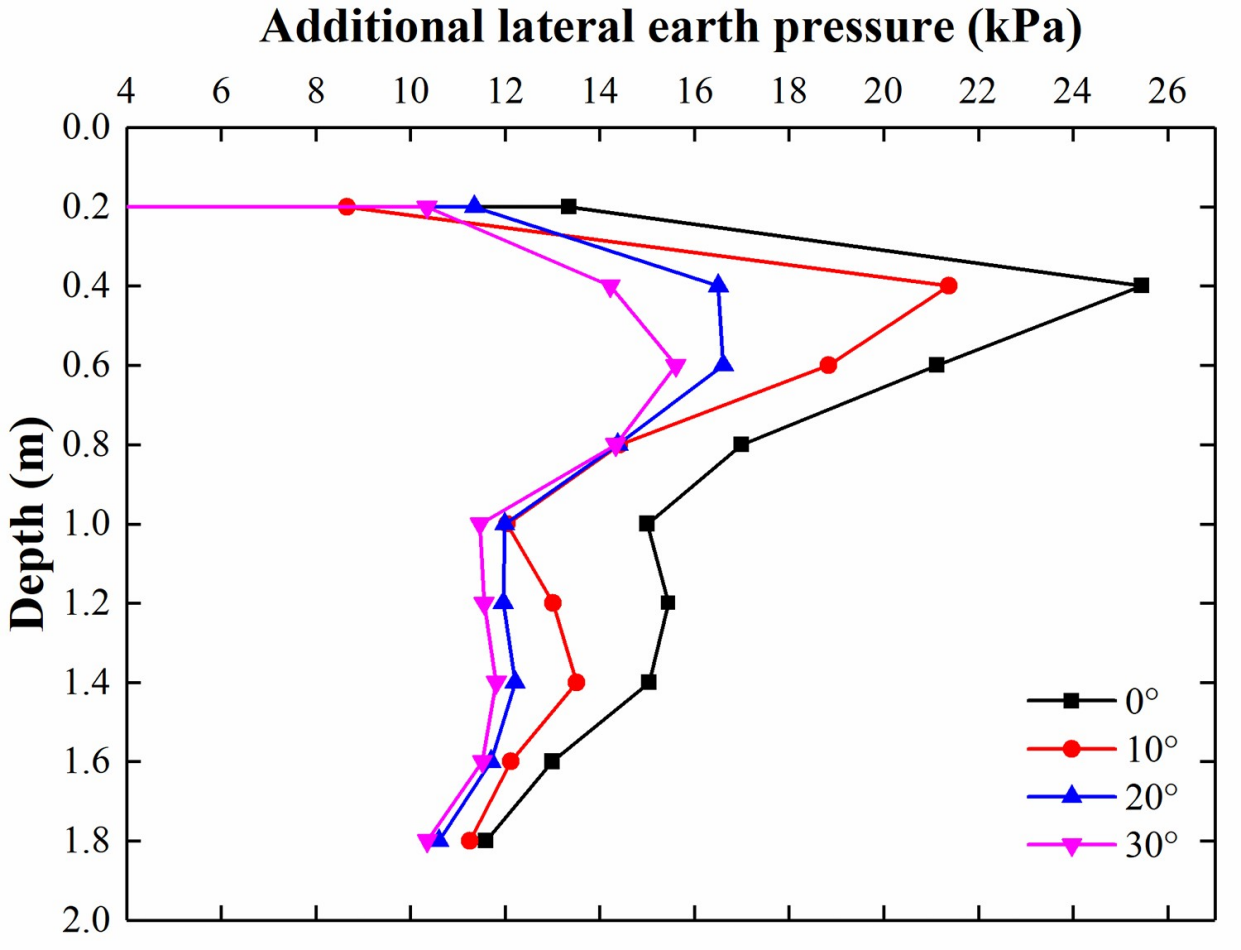
Comparison of the lateral earth pressure for different wall-soil friction angles.

In addition, the friction angle of the wall and soil is one of the most important factors affecting the earth pressure acting on the retaining structure. Fig 34 illustrates the distribution of the lateral earth pressure for different wall-soil friction angles. Fig 34 shows that the lateral earth pressure decreased with the increasing wall-soil friction angle and the variation of the lateral earth pressure decreased with the increasing friction angle, because the principal stress axis of the soil deflects with increasing friction angle, and the soil arch effect occurs on the interface between wall and soil. Thus, the lateral earth pressure decreases with the increasing wall-soil friction angle.

From the parametric study, we can conclude that the length of the pile can affect the location of the additional earth pressure caused by the pile and that the elastic modulus of the pile can affect the magnitude of the additional earth pressure caused by the pile. In addition, the lateral earth pressure decreases with the increasing wall-soil friction angle.

## 5. Conclusions

A series of model tests were conducted to investigate the additional lateral earth pressure acting on the retaining structure adjacent to the CFRLP and NG. Two testing procedures, including applying a load to the foundation and rotating the retaining structure along its toe, were considered. In addition, a parametric study was conducted in this paper. Based on the limited number of tests and FEM analysis, the following conclusions were obtained:

1. The additional lateral earth pressure acting on the retaining structure adjacent to CFRLP is less than that of the NG in the depth of the flexible reinforcement area. There are three reasons for the above phenomenon: the decrease in the load-sharing ratio in the soil, the blocking of the piles and the decrease in the surcharge load transfer ability of the soil.
2. Compared with NG, the CFRLP experiences a smaller normalized height of application of the lateral earth pressure. This indicates that the CFRLP blocked the horizontal diffusion of the load and had a stronger ability than the NG to transfer the surcharge load to the deep soil.
3. When rotating the retaining structure, the lateral earth pressure acting on the upper part of the retaining structure shows limited reduction once the displacement at the top of the retaining structure is greater than 8 mm, whereas that acting on the lower part of the retaining structure continues to decrease with the increased displacement (or rotation amount). This may be because with increasing rotation of the retaining structure, the soil adjacent to the upper part of the retaining structure enters the active limit state.
4. The parametric study shows that the length and the elastic modulus of the pile can greatly affect the location and the magnitude of the additional lateral earth pressure caused by the pile. The soil arch effect caused by the increase in the wall-soil friction angle will greatly reduce the value of the lateral earth pressure.

## Supporting information

S1 Dataset Experiment data for all cases. (XLS)
